# Pharmacometabolomics for the Study of Lipid-Lowering Therapies: Opportunities and Challenges

**DOI:** 10.3390/ijms24043291

**Published:** 2023-02-07

**Authors:** Erica Gianazza, Maura Brioschi, Ada Iezzi, Giuseppe Paglia, Cristina Banfi

**Affiliations:** 1 Monzino Cardiologic Center, IRCCS, 20138 Milan, Italy; 2 Division of Pharmacy, IEO European Institute of Oncology IRCCS, 20141 Milan, Italy; 3 School of Medicine and Surgery, University of Milano-Bicocca, 20854 Vedano al Lambro, Italy

**Keywords:** metabolomics, lipid-lowering drugs, statins, atherosclerotic cardiovascular disease

## Abstract

Lipid-lowering therapies are widely used to prevent the development of atherosclerotic cardiovascular disease (ASCVD) and related mortality worldwide. “Omics” technologies have been successfully applied in recent decades to investigate the mechanisms of action of these drugs, their pleiotropic effects, and their side effects, aiming to identify novel targets for future personalized medicine with an improvement of the efficacy and safety associated with the treatment. Pharmacometabolomics is a branch of metabolomics that is focused on the study of drug effects on metabolic pathways that are implicated in the variation of response to the treatment considering also the influences from a specific disease, environment, and concomitant pharmacological therapies. In this review, we summarized the most significant metabolomic studies on the effects of lipid-lowering therapies, including the most commonly used statins and fibrates to novel drugs or nutraceutical approaches. The integration of pharmacometabolomics data with the information obtained from the other “omics” approaches could help in the comprehension of the biological mechanisms underlying the use of lipid-lowering drugs in view of defining a precision medicine to improve the efficacy and reduce the side effects associated with the treatment.

## 1. Introduction

Atherosclerotic cardiovascular disease (ASCVD) is considered the leading cause of death globally [1]. Established lipid-lowering therapies are available, which reduce low-density lipoprotein cholesterol (LDL-C), preventing ASCVD and mortality, but they are still insufficient to bring a halt to the ASCVD epidemic. Strong efforts have been made in recent decades to fully characterize the mechanism of action of these drugs, their pleiotropic effects, and their side effects using both hypotheses-driven and “omics” approaches, such as genomics, proteomics, and metabolomics.

Several proteomics studies have contributed substantially to better understanding the profiles of cholesterol-lowering drugs, aiming to elucidate the mechanism of action by drug–protein interaction and to discover multiple drug-specific targets in treated patients by monitoring the pharmacological effect [2,3,4].

The integration of proteomics and metabolomics has led to assessing both protein and metabolite changes improving the understanding of pathophysiological mechanisms and the discovery of novel biomarkers for future personalized drug therapy with higher efficacy and safety. Successful metabolomic studies have been recently performed in different contexts, such as for the evaluation of the associations of plasma metabolome with subclinical atherosclerosis in diabetes patients from the Diabetes Heart Study [5]. 

Metabolomics allows the quantitative detection of multiple small molecule metabolites and lipids in biological systems and the investigation of the alterations in metabolic pathways and networks, thus providing information on the mechanisms underlying the beneficial effects and adverse metabolic consequences of a drug [6]. The quantitative measurement of the dynamic metabolic responses of a person to pathophysiological stimuli, drug therapy, or other interventions is often defined as metabonomics and involves the characterization of the changes of metabolic products in biological fluids and tissues following disease processes, environmental factors, drugs, and gut microflora [7]. The difference between metabonomics and metabolomics is minimal and the terms are often used interchangeably because the analytical procedures are the same.

Considering that the metabolome is both impacted by genetic background and environmental exposure, it provides a more specific description of the phenotype. Indeed, the metabolic profile is strongly influenced by both pathophysiological status and external perturbation such as specific drug treatment [8].

This intrinsic characteristic of the metabolome leads to the development of a specific metabolomics branch, pharmacometabolomics, which is now viewed as a complementary technique to genomics, transcriptomics, and proteomics for the therapeutic evaluation of specific drug products. Pharmacometabolomics or pharmacometabonomics contributes to a comprehensive understanding of the drug effects by also considering influences from a particular disease, environmental factors, diet, and concomitant pharmacological treatments [9]. This approach can be used to better understand the pharmacokinetic profile of a drug or to evaluate the metabolite levels after a pharmaceutical treatment, thus clarifying the mechanisms underlying the variations in response to therapy. In addition, it can provide potential unique signatures useful to stratify patients based on their metabolic heterogeneity within a specific disease state and to predict individual therapeutic responses [10].

Through pharmacometabolomics is possible to improve the efficacy and minimize the side effects associated with a treatment [11]. Indeed, in the last few years, the focus on drug therapy has moved further toward a personalized approach. Personalized therapy is very important in medical treatment, and it first of all requires the ability to recognize a different response to a specific drug in individuals [12]. In this respect, longitudinal studies using pharmacometabonomics have become a valuable tool to examine individual metabolic responses and, consequently, to direct toward a correct personalized medicine [13,14,15]. Thus, thanks to the recent evolution of the metabolomic field, metabolomic-based platforms are now also employed during the early phases of drug discovery, from target engagement to the elucidation of the mechanisms of action and discovery of markers for therapies monitoring, and have the potential to accelerate drug development [16]. 

The highly dynamic profiles of metabolites and their extreme chemical diversity present a challenge to research; thus, there is no single analytical technique that is able to cover the entire range of metabolites in a complex biological sample providing a comprehensive metabolomic analysis [17]. Among the analytical platforms employed in metabolomic analysis, nuclear magnetic resonance (NMR) and mass spectrometry (MS) are the most widely used. Due to higher sensitivity and throughput, MS is often applied both in untargeted and targeted metabolomics in combination with previous separation techniques, such as liquid chromatography (LC), gas chromatography (GC), and capillary electrophoresis (CE), that enhance the analytical capabilities of MS, improving the metabolite coverage of the analysis and facilitating the identification of the numerous compound classes [18]. Therefore, it is necessary to combine different separation procedures to cover a wide portion of the metabolome, even if this is time-consuming and data processing is more complicated. One of the main analytical methods used for metabolite analysis is LC-MS, and in particular, in recent years, the application of two-dimensional (2D) LC has grown, allowing higher sensitivity of the analysis, an improvement in compound separation when single-dimension separation is difficult, and, consequently, a significant increase in the compound number measured [17]. The metabolomic analysis can be either untargeted or targeted, depending on the research question and several other factors, including the classes, stability, and chemical properties of the metabolites of interest, as well as the appropriate analytical accuracy. Metabolomics can provide an untargeted identification of hundreds to thousands of metabolites simultaneously within a sample, achieving qualitative data as relative intensities of the metabolites associated with a particular pathophysiological status [19]. On the other hand, a targeted analysis allows the measurement of specific known compound classes using stable isotope labelled internal standards, thus providing absolute quantitative data. The most commonly used technique for targeted analysis is selected reaction monitoring (SRM) or multiple reaction monitoring (MRM) when multiple proteins are measured during a single MS analysis.

The rapid and continuous development of high-throughput analytical strategies and more robust bioinformatic tools will improve the use and integration of metabolomics into the biomedical research to discover new diagnostic and prognostic measures and drugs. 

This review summarizes the currently available metabolomics studies performed to address the effects of lipid-lowering therapies from widely used statins and fibrates to proprotein convertase subtilisin/kexin type 9 (PCSK9) inhibitors or nutraceutical approaches.

## 2. Methodological Approaches for Pharmacometabolomics and Pharmacolipidomics

Metabolomics can be applied to multiple biological matrices, such as tissues, cells, and biofluids. Plasma and serum are easily accessible and the most widely used matrices in human metabolomic clinical studies [20,21], together with urine samples which are commonly used for both human and animal metabolomics studies. However, metabolomics has also found several applications with less used biosamples, such as feces, saliva, culture medium, cells, or tissues [22], which can likewise provide interesting information on biological functions in health and disease. Feces, for example, are of increasing interest in metabolomics studies, because they reflect the metabolic association between the host and its intestinal microbiota [23,24]. Instead, even though tissues are invasive sample types, their analysis is also important, as they describe the metabolic changes that occur as a result of a disease. Tissue composition is often inhomogeneous in composition, thus increasing biological variability which should be always considered during sample collection and treatment. Indeed, pre-analytical handling steps are an important aspect of metabolomics study design, because accurate sample collection, processing, and storage are crucial to preserving sample integrity and quality. For this reason, guidelines and specific standard operating procedures for the pre-analytical handling of samples are required prior to initiating a metabolomic study to increase the metabolite recovery and stability for further metabolic investigation [23].

Furthermore, there is a plethora of literature on extraction procedures for metabolomics but there is no optimal procedure because it depends on the molecular targets given the complexity of biological matrices, metabolite enzymatic turnover rates, or the need to enrich low-abundance compounds [25].

Metabolomics is a technology-driven discipline, like all the other “omics” approaches, strongly based on new developments in analytical techniques, instrumentation, software, and methods for data analysis. 

As mentioned above, MS and NMR are the most widely used technologies for metabolomics, allowing the qualitative and quantitative analysis of metabolites in biological samples. MS is often coupled to other separation techniques such as GC and LC to better resolve and characterize the metabolome, providing analytical platforms able to separate ions beyond the mass-to-charge ratio (*m*/*z*).

**Figure 1:**
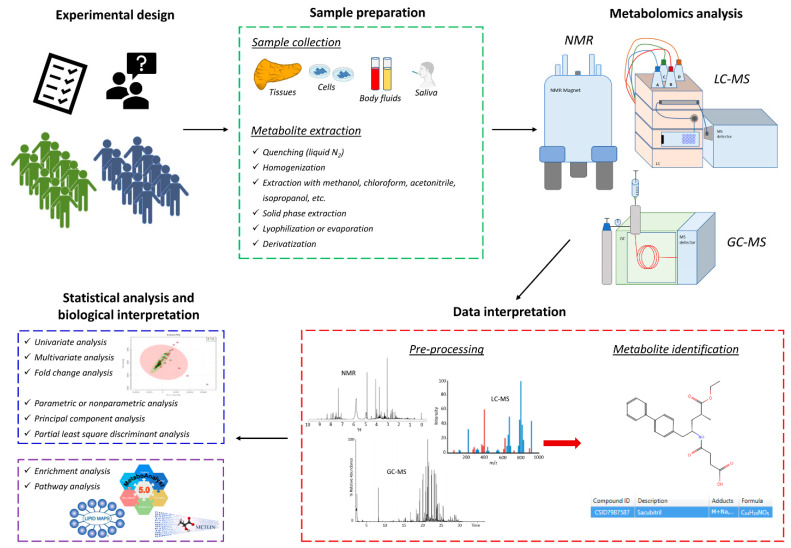
Schematic workflow of metabolomic studies. Several steps are involved from experimental design and biological sample preparation to data analysis, processing, and interpretation. Nuclear magnetic resonance (NMR) spectroscopy and mass spectrometry (MS) are the main analytical technologies used in metabolomics research. GC-MS, gas chromatography-MS; LC-MS, liquid chromatography-MS.

Therefore, most of the metabolomics studies have been performed using GC-MS, LC-MS, or NMR, which are briefly described below since details of the technical aspects have been clearly reviewed elsewhere [26,27]. Of note, none of them allow the complete identification and quantification of all metabolites in biological samples, because they have specific advantages and disadvantages [28]. A typical workflow for metabolomic experiments is reported in Figure 1.

Lipidomics is considered a subfield of metabolomics because it gives information about the global lipid profile of a biological system that can be closely associated with the other metabolic pathways in the understanding of all mechanisms mediating statin effects. Similarly to metabolomics, lipids can be divided into several classes based on their different structural properties, and many analytical strategies have been developed, including targeted, untargeted, and shotgun lipidomics [29,30]. Lipidomics employs similar analytical techniques to metabolomics, even if MS-based techniques are still the most widely used approach by a direct analysis of the sample or following a lipid extraction and separation procedure [31]. 

Due to the advances in MS, lipidomics has grown in the last decade providing both qualitative and quantitative data on multiple lipid categories whose changes can be involved in many metabolic diseases, such as ASCVDs and diabetes. The study of lipids and their metabolic pathways has significant potential for finding biomarkers and developing innovative therapeutic targets for real-world biological questions [31]. 

Recently, high-density lipoprotein (HDL) lipidome was investigated by NMR in patients with prediabetes and compared to profiles from normoglycemic subjects and patients with established type 2 diabetes [32]. Significant qualitative and quantitative alterations in HDL lipidome, potentially with proatherogenic properties, were observed in prediabetic patients compared to normoglycemic individuals. These changes in the lipid composition of HDL were qualitatively similar but with quantitative levels less severe than those measured in diabetic patients. Therefore, progressive changes in HDL lipidome from healthy subjects to patients with prediabetes and type 2 diabetes was reported, demonstrating that lipidomics helps to study the metabolic pathways underlying the progression to diabetes and to identify patients with high risk of developing CVD. Although none of the patients involved in this study were taking lipid-lowering drugs or other therapies known to affect lipid metabolism, this study highlighted significant variations in lipid composition of serum lipoproteins [32].

It has become clear that impaired metabolism and lipid composition of serum lipoproteins is critical for the pathogenesis of CVDs. The beneficial effect of lipid-lower therapies has been extensively studied in biomedical research demonstrating an improvement of circulating lipid levels and generally lipid metabolism. In this regard, pharmacolipidomics helps to explore the underlying mechanisms of lipid metabolism and its regulation by lipid-lower therapies in pathological conditions. Moreover, it could be a powerful approach for risk stratification of patients, discovery of disease biomarkers, treatment evaluation monitoring of therapeutic responses of multiple lipid classes, as well as indications for future personalized medicine.

This important role of pharmacolipidomics was clearly demonstrated in a lipidomic analysis performed to study the association of coronary artery stenosis with atherogenic (non-HDL) and atheroprotective (HDL) lipid profiles in patients with coronary heart disease at various stages and compared with subjects with normal coronary arteries [33]. Patients with different grades of coronary artery stenosis were separated from subjects with normal coronary arteries in the atherogenic model, and the severe stage was also distinguished from both mild and moderate with significant power. Instead, in the atheroprotective model, only the differentiation between patients with mild and severe coronary artery stenosis was statistically significant. Therefore, the study of lipid species in lipoproteins provides the possibility to identify early markers of the onset of cardiac diseases and determine the best therapeutic approach for patients [33].

### 2.1. Nuclear Magnetic Resonance (NMR)

NMR is an extensively used analytical platform in metabolomics studies, addressing the specific metabolic changes associated with mechanisms of action or toxic effects of several drugs [22].

For most ^1^H-NMR-based metabolomics studies, sample preparation requires the addition of a deuterated buffer to the blood or urine samples to adjust the pH and provide the necessary lock signal [34]. Indeed, pH adjustment is particularly relevant in urine or saliva, which are particularly sensitive to inter-individual pH changes. This approach offers the advantage of easy sample preparation, the ability to obtain and absolute quantitate metabolites, high reproducibility, and, of note, non-destructiveness. The only disadvantage of this approach is in terms of sensitivity, which is 10 to 100 times less than MS-based techniques. Moreover, it is highly reproducible and, therefore, more suitable for large-scale metabolomics studies than MS-based methods, and can be used to detect and characterize sugars, organic acids, alcohols, polyols, and other highly polar compounds, which can be more difficult to detect with MS-based approaches [28]. 

NMR-based metabolomics and lipidomics have been extensively applied to find specific patterns for the diagnosis and prognosis of different human diseases, such as ASCVDs [35]. NMR was recently applied in a cross-sectional study to evaluate plasma metabolomics and lipidomics in atherosclerosis according to the presence of type 1 diabetes or previous preeclampsia [36]. Significant differences were reported according to the presence of diabetes or preeclampsia, which is known to have implications for future cardiovascular disease (CVD) events, and it has been shown that circulating levels of phosphatidylcholine, free cholesterol, saturated fatty acids, and w-7 fatty acids were independently associated with preclinical carotid atherosclerosis.

In another recent large prospective cohort study, metabolomic and lipidomic profiles and their relationship to infection burden during the first year of life were characterized by NMR [37], demonstrating that infants with a higher infection burden had proinflammatory and proatherogenic plasma metabolomic and lipidomic profiles that in adults suggest an enhanced risk of CVD, obesity, and type 2 diabetes. Therefore, the study of the cardiometabolic associations with common infections and inflammation in early life is very important to perform a risk stratification and develop targeted interventions to prevent CVD implications.

Since ACVDs are the major cause of death in patients with type 2 diabetes mellitus, ^1^H-NMR-based lipidomic technology was also applied to evaluate compositional features of the HDLs in healthy subjects with normal coronary arteries, diabetic patients with normal coronary arteries, and patients with acute coronary syndrome [38]. Significant lipid alterations in HDL were observed in diabetic patients compared to controls, an atherogenic pattern that is further aggravated in patients with established coronary heart disease. Similarly, NMR was applied to study serum metabolome of subclinical atherosclerosis, measured using ankle brachial index (ABI), in people with type 2 diabetes, compared with the profile for symptomatic CVD in the same cohort of individuals [39]. This study revealed that glycolysis-related metabolites, fluid balance molecules, and inflammation markers were independently associated with ABI and symptomatic CVD. 

All these studies demonstrate the potential of NMR-based lipidomics and metabolomics for disease biomarker discovery in complex biological samples in pathophysiological conditions. 

### 2.2. Gas Chromatography-Mass Spectrometry (GC-MS)

GC-MS is the most standardized method for metabolomic studies, based on more than 50 years of analyses, resulting in an increasing number of complete publicly available libraries under standardized conditions of 70 eV electron ionization energy, such as the NIST 14 Mass Spectral Library collection of the U.S. National Institute of Standards and Technology (NIST) containing mass spectra for 242,477 unique compounds, partially characterized also in terms of retention times, for a better performance of metabolite identification. Despite the notable breadth, sensitivity, and specificity of metabolite detections, GC-MS requires a derivatization step performed under very mild conditions, to decrease boiling points and increase the stability of compounds for GC-MS analysis [25].

Recently, lipid peroxidation aldehyde metabolites were compared in cardiovascular patients and healthy controls using a targeted and untargeted metabolomics approach based on an advanced combined derivatization/solventless extraction procedure from plasma followed by GC-MS [40]. The targeted GC-MS approach showed that hexanal, malondialdehyde and 4-hydroxynonenal are significantly higher in CVD patients, while untargeted GC-MS also reported higher levels of hexanal and lower levels of citral in CVD patients compared to control subjects.

Since type 1 diabetes is associated with premature CVD, GC-MS was also applied to measure plasma concentrations of free fatty acids in diabetic patients and healthy controls, highlighting specific changes in lipid metabolism of patients that could have consequences for inflammation, cellular function, and oxidative stress management [41].

Therefore, GC-MS has always been considered one of the most efficient and robust analytical platforms for metabolomics and lipidomics research, and is complementary to LC-MS so that it can be used to obtain full coverage of metabolite species in biological studies. 

### 2.3. Liquid Chromatography-Mass Spectrometry (LC-MS)

Alternative to GC, LC is largely used for metabolomic and lipidomic studies in combination with high-resolution mass spectrometers as well as tandem MS for targeted analysis to improve specificity, taking advantage of different selectivities in LC separation and the high sensitivity in MS detection. Thanks to technological advances, thousands of features can be detected. One of the most-used separation techniques is reversed phase for the analysis of mid- to non-polar metabolites and it is often used for lipid analysis, while small polar metabolites, such as amino acids, carboxylic acids, sugars, etc., can be resolved using hydrophilic interaction liquid chromatography separation [17]. 

However, misidentification can be present due to overlapping compounds with similar molecular weight (<5 ppm), in source degradation products, or the presence of isomeric and isobaric species that cannot be discriminated by MS, except for instruments equipped with ion mobility separation [42]. Differently from NMR, untargeted LC-MS and GC-MS provide relative quantities with respect to a reference sample, because peak intensities are not directly proportional to concentration due to differential ionization efficiencies of metabolites in complex mixtures [43].

LC-MS is a very sensitive and specific technique for lipidomic analysis and has been extensively applied in heart biology for the discovery of potential biomarkers. Recently, a sequential lipid profiling from acute to chronic heart failure was performed in mouse and human samples by an untargeted LC-linear trap quadrupole orbitrap MS [44]. Both tissue and plasma lipidomic profiles were acquired and compared to identify potential lipid markers for heart failure. Multivariate analysis showed distinct cardiac lipidomic patterns between healthy and ischemic patients, including significantly reduced glycerophospholipids in the ischemic heart especially phosphatidylethanolamines that were considered the main class of ischemia biomarkers. Phosphatidylethanolamines levels were instead significantly enhanced in tissues and plasma from risk-free mice in chronic myocardial infarction, thus suggesting a possible physiological cardiac remodeling. In addition, a reduced mitochondrial function associated with several altered lipid levels seemed to be an early marker of acute heart failure. The fold change analysis reported site-specific lipid metabolites and inter-organ lipidomic patterns that were significantly associated with acute and chronic heart failure, thus demonstrating a strong pathological lipid remodeling [44].

In a previous study, using LC-MS in SRM mode, the same authors absolutely quantified sphingolipid mediators in ischemic human hearts, and measured them also in murine spleen and heart as an integrative approach, as well as in plasma samples, to investigate the role of sphingosine-1-phosphate interaction with its receptors in the transition of acute to chronic heart failure [45]. They demonstrated that the early measurement of sphingosine-1-phosphate levels could be a promising and effective strategy for heart failure treatment. 

Therefore, high-throughput quantitative lipidomics using LC-MS is a powerful approach to study lipid signaling in molecular and cellular pathways, because it provides the possibility to discover candidate diagnostic/prognostic markers or new therapeutic targets for cardiac protection in clinical translation. 

Another recent example of LC-MS application concerns the characterization of the lipidome of the main phospholipids and sphingolipids species in HDL subfractions to investigate their association with premature coronary heart disease or metabolic syndrome in families where a low HDL cholesterol level prior to statin treatment predisposed to premature coronary heart disease [46]. The lipidome of phosphatidylcholines, lysophosphatidylcholines, and sphingomyelins in HDL subfractions was associated with cardiometabolic disorders. Distinct fatty acid compositions of HDL phospholipids were observed to be characteristic of both metabolic syndrome and premature coronary heart disease.

## 3. Lipid-Lowering Therapies and Metabolomics

**Figure 2:**
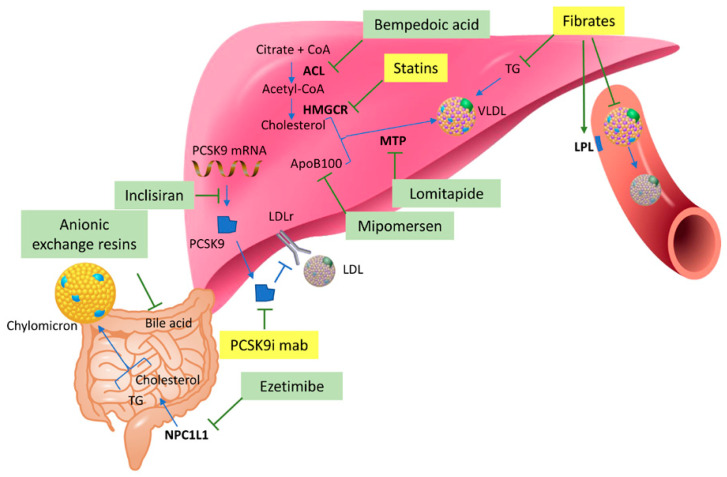
Sites and targets of lipid-lowering therapies. Diagram of the mechanisms of action of the principal lipid-lowering drugs including those drug classes that have not yet been the object of pharmacometabolomic studies. Drug classes analyzed in metabolomic studies are highlighted by yellow boxes. Statins reduce cholesterol synthesis through 3-hydroxy-3-methyl-glutaryl-coenzyme A reductase (HMGCR) inhibition. Fibrates are peroxisome proliferator-activated receptor alpha (PPARα) activators, able to increase Lipoprotein lipase activity and globally reduce triglycerides (TG) levels. Reduction of proprotein convertase subtilisin/kexin type 9 (PCSK9), responsible for the degradation of low-density lipoprotein receptor (LDLR), can be achieved with monoclonal antibodies or Inclisiran, a siRNA specific for PCSK9 that prevents translation of PCSK9 messenger RNA. Other drug classes without pharmacometabolomic studies are reported in green boxes. Bempedoic acid is a potent inhibitor of adenosine triphosphate (ATP) citrate lyase (ACL), a cellular enzyme responsible for the production of precursors for fatty acid and cholesterol synthesis. Lomitapide is an inhibitor of microsomal triglyceride transfer protein (MTP), an enzyme responsible for the synthesis of very low-density lipoproteins in the liver and chylomicrons in the intestine. Anionic exchange resins are bile acids sequestrants. The depletion of the bile acid pool stimulates the conversion of cholesterol to bile acid, reducing intracellular cholesterol in hepatocytes. Ezetimibe selectively inhibits intestinal cholesterol absorption, inhibiting the cholesterol transport protein Nieman Pick C1-like 1 protein (NPC1L1) in the intestine. ApoB100, apolipoprotein B100; LDL, low-density lipoprotein; LDL-r, LDL receptor; mab, monoclonal antibody; CoA, coenzyme A.

Due to the important role of dyslipidaemia in the occurrence of atherosclerotic CVD, different approaches have been developed to lower LDL-cholesterol to improve CVD outcome. Despite the well-demonstrated benefits of statins, a proportion of patients does not reach the target levels of cholesterol recommended by guidelines, mainly due to low compliance associated with side effects [47,48]. Nowadays, alternative cholesterol lowering approaches have been developed to obtain better results both in terms of efficacy and tolerability, and they are often used in combination with statin. Fibrates act through the activation of peroxisome proliferator-activated receptor-α (PPAR-α) to modulate lipid and lipoprotein metabolism. One of the most recent classes of lipid-lowering therapy is represented by the PCSK9 inhibitors involved in the control of LDL receptor. The reduction of cholesterol absorption in the intestine can be obtained with ezetimibe interacting with the Niemann-Pick C1-like protein 1 (NPC1L1). Bile acid sequestrants indirectly reduce cholesterol that is needed by the liver to synthesize novel bile acids. Microsomal TG transfer protein (MTP) inhibitor, Lomitapide, prevents very-low-density lipoprotein (VLDL) formation in the liver and chylomicron formation in the intestine, acting on the transfer of triglycerides and phospholipids to Apolipoprotein B (ApoB). The antisense oligonucleotide, mipomersen, reduces the liver translation of the ApoB protein. The novel Bempedoic acid (ETC-1002) acts as an inhibitor of adenosine triphosphate citrate lyase (ACL), involved in the production of precursor of cholesterol and fatty acids [49]. Novel approaches have been also developed to reduce angiopoietin-like protein 3 (ANGPTL3) with a blocking antibody or antisense nucleotide, or to reduce mRNA levels of apolipoprotein CIII [48]. Although the pharmacological mechanisms of lipid-lowering drugs have been extensively studied, their metabolism-regulating effects and adverse effects have not been fully elucidated, especially after long-term treatments [50]. Indeed, as shown in Figure 2, describing the sites and targets of the main lipid-lowering drugs or drug classes, it is evident that, at the moment, only few of them have been studied from a metabolomic point of view in preclinical or clinical studies.

### 3.1. Statins

Statins are a potent group of lipid-lowering drugs, also known as 3-hydroxy-3-methyl-glutaryl-coenzyme A (HMG-CoA) reductase inhibitors, that can lower the levels of LDL-C in the blood. They are the primary cholesterol-lowering medications that work by reducing the LDL-C production inside the liver by competitively inhibiting the enzyme HMG-CoA reductase, which converts HMG-CoA to mevalonate formed during cholesterol synthesis [51]. Consequently, statins have proven to be an effective and efficient therapeutic way in the primary and secondary prevention of coronary heart diseases. In patients with very high LDL-C levels and a high risk of cardiovascular diseases, statins reduce the hardening and narrowing of the arteries and stabilize the plaques on blood vessel walls when atherosclerosis is already present. Moreover, they improve endothelial function, decrease inflammation status and oxidative stress, and prevent thrombogenic response [52].

A lifestyle modification through a cholesterol-lowering diet and physical exercise is recommended as a first choice but when the cholesterol levels continue to stay high after healthy lifestyle changes are implemented, statins can be helpful. The intake of statins depends on cholesterol levels and other cardiovascular risk factors that must be carefully considered and examined. Various forms of statins are commercially available and they are often prescribed in combination with other drug agents, such as ezetimibe, colesevelam, fibrates, or niacin [53,54], to increase their beneficial effects for the treatment of hyperlipidemia. However, statins are prescribed on a long-term basis and are well-tolerated by most people, but they can also have serious adverse effects in some cases, such as liver damage, muscle problems, neuropathy, and increased blood sugar that can lead to type 2 diabetes mellitus [55]. Moreover, statins may also undergo drug–drug interactions with other drugs, including agents that are commonly used for the treatment of cardiovascular diseases, leading to pharmacokinetic alterations and increased risk of myopathy, hepatotoxicity, and immune effects [56]. Therefore, it is essential to study in depth the pharmacological response to statins in patients to understand better the real efficacy and safety of statins for definitive future guidelines.

#### 3.1.1. Statin Response Variability

Statins have a variety of pleiotropic effects which are not yet fully understood. Characterizing the metabolic profiling following statin treatment provides further information about metabolic pathways involved in the pleiotropic effects of statins. Recently, Silva et al. [57] performed the first comprehensive evaluation of the metabolic signature of simvastatin treatment in a large population-based study, demonstrating that simvastatin shows several pleiotropic effects in the participants with statistically significant changes in multiple metabolite concentrations, affecting not only lipids, but also amino acids, peptides, nucleotides, carbohydrates, co-factors, vitamins, and xenobiotics. They identified more than 300 “novel” metabolites previously unpublished in association with simvastatin treatment, in particular short-chain acyl-carnitines and amino acids, thus reporting a more complex metabolic signature of simvastatin treatment compared to what was thought [57]. These “novel” metabolites are already known in the literature, but were reported for the first time in this study in the participants that used simvastatin.

In addition, pharmacometabolomics can help to study the interindividual variation in the response to statins [58]. It is important to evaluate such variability, which may affect drug efficacy and toxicity, before the drug administration in patients. In the literature, several pharmacometabolomic studies focus on the characterization of the metabolic profiles of patients before treatment to stratify them as “responders” or “non-responders” to a specific therapeutic intervention [22].

The identification of predictive pre-treatment metabolic markers could be very useful in clinics to define individual variation, improve LDL-C lowering, and minimize drug toxicity [12]. 

For this reason, a pharmacometabonomic approach was applied to predict metabolic phenotypes and pharmacokinetic parameters of atorvastatin in healthy volunteers to investigate the individual differences in drug response without any prior knowledge of the genomic profile [12]. The authors measured the levels of metabolites in pre-dose baseline plasma samples from 48 healthy volunteers using a GC-MS-based global metabolic profiling and quantified the atorvastatin levels in plasma at various time points after oral administration using an LC-MS/MS system operating in MRM mode. The untargeted metabolite profiling was performed on baseline plasma samples of all participants leading to the identification of several peaks, including amino acids, organic acids, carbohydrates, fatty acids, steroids, and many other compounds. In addition, a high degree of individual variation regarding pharmacokinetic responses was reported measuring the plasma concentration of atorvastatin. At this point, the group of participants was randomly divided into a training set and a prediction set for the subsequent multivariate statistical modeling, which was applied to screen potential markers of individual diversity by correlating endogenous metabolites in pre-dose plasma with pharmacokinetic parameters [12]. Using the baseline metabolic profiles of the subjects in the training set, a prediction model of multiple features was created and correctly predicted the pharmacokinetic parameters of the healthy volunteers in the prediction group. Endogenous molecules showed a good correlation with pharmacokinetic parameters, and in particular tryptophan, alanine, arachidonic acid, 2-hydroxybutyric acid, cholesterol, and isoleucine were considered as potential markers for predicting individual differences among the volunteers. 

Indeed, it has been demonstrated that monocarboxylate transporters mediate the transport of endogenous aromatic amino acids such as tryptophan, tyrosine, and phenylalanine across the plasma membrane, as well as metabolically important monocarboxylates in the intestine, such as benzoic acid, short chain fatty acids, and drugs with monocarboxylate structures [59,60]. Since atorvastatin is a monocarboxylic acid, it has been reported its intestinal absorption by monocarboxylate transporters [61], whose inhibition causes a reduction of drug bioavailability. Thus, the potential markers identified in this study, including tryptophan, tyrosine, phenylalanine, and the monocarboxylic acid 2-hydroxybutyric acid, could play an important role in predicting atorvastatin pharmacokinetics, because they affect the activity of the intestinal monocarboxylate transporters and compete with the atorvastatin immediately after its administration.

Using pre-drug plasma metabolic profiles, this pharmacometabonomic approach could help to effectively predict individual variances in pharmacokinetics before atorvastatin administration and stratify individuals into subgroups that are more likely to be responsive to drug therapy [12]. Consequently, it will be possible to define a better individualized statin therapy, which means the optimal drug dosing, avoiding adverse drug reactions by means of the characterization of the metabolic profile of each individual. Of course, the investigation of biological fluids other than plasma, as well as increasing the coverage of the metabolome by using other analytical platforms (e.g., LC-MS and NMR) could be useful to obtain an even more accurate predictive pharmacometabonomic model.

Nowadays, the field of metabolomics includes multiple analytical platforms and bioinformatics tools for mapping pathways implicated in disease and individual variation in response to drugs. 

Krauss et al. carried out many studies with complementary metabolomics platforms to characterize the global effects of simvastatin, a semisynthetic derivative, on metabolism and identify potential markers of the variability in plasma low-density lipoprotein (LDL) response to statin treatment [58]. They applied three different metabolomic and lipidomic platforms to analyze plasma samples obtained from the Cholesterol and Pharmacogenetic (CAP) study, which is a 6-week non-randomized clinical trial of 40 mg/day simvastatin treatment in a group of African American and Caucasian volunteer subjects [62]. 

Over 300 lipid species within eight lipid classes were measured by a targeted GC-based lipidomic platform, demonstrating that baseline cholesterol ester and phospholipid metabolites correlated with LDL-C response to treatment [63]. Moreover, they showed statin-induced changes in C-reactive protein, a marker of inflammation and a potential predictor of CVD risk, that were significantly correlated with baseline concentrations of plasmalogens. Since plasmalogens are involved in inflammatory processes, they assumed a role of plasmalogen metabolism in modifying the well-known anti-inflammatory effects of statins and resulting in inter-individual variation [63]. 

A targeted GC-MS sterol and bile acid metabolomics platform also confirmed the potential role of intestinal bile acid metabolism in modulating simvastatin efficacy through effects on tissue transport [64], because a single nucleotide polymorphism (SNP) in the gene encoding a transporter in the liver and intestine (i.e., *SLCO1B1* encoding OATP1B1) has been associated with both statin LDL-C lowering efficacy and risk of statin-associated myopathy [65,66]. Bile acids and related metabolites regulate the expression of OATP1B1 transporter; thus, using their baseline levels it is possible to better understand the competitive interactions with the drugs transported by OATP1B1, and consequently to allow a prediction of the pharmacokinetic response to statins [12]. Indeed, the competition between simvastatin and bile acids for this transporter markedly affects the pharmacokinetics of simvastatin, and can, thus, greatly impact both the efficacy and safety of this drug. Finally, using untargeted metabolomics with a GC-time of flight (TOF) instrument, the same authors measured in the full range responders over 300 metabolites of intermediary metabolism, and they observed the effects of statins on amino acids and their degradation products, with changes in cystine, glutamine, urea cycle intermediates, and the dibasic amino acids ornithine, citrulline, and lysine [67]. The alterations in citrulline and ornithine concentrations reflect an increased flux through the urea cycle, thus suggesting a change in amino acid degradation. Moreover, lower baseline levels of xanthine and reduced purine degradation stimulated a more robust nitric oxide synthase (NOS) signaling. Since NOS catalyzes the production of nitric oxide (NO) from arginine, and it is also known that statins increase the expression and activity of endothelial NOS, the upregulation of NO due to the statin treatment confirmed an increased production of NO with beneficial effects on endothelial function [68]. This evidence proves the important therapeutic effect of statins in cardiovascular diseases such as atherosclerosis, heart failure, and hypertension [69]. Following exposure to simvastatin, a strong correlation between alpha and gamma-tocopherol (vitamin E) and cholesterol metabolism was also reported in all responders, and a significant decrease was found in their plasma levels [67]. Their reduction in plasma was accompanied by increased tocopherol content of LDL particles because statin treatment promotes the LDL capacity to transport lipid-soluble antioxidant vitamins [70] giving greater resistance to these particles to oxidative stress and reducing their atherogenic potential. In addition to having antioxidant properties, alpha tocopherols modulate, in a concentration-dependent mode, the LDL receptor binding activity, which is well known to have a role in plasma cholesterol homeostasis, as already demonstrated before by Pal et al. [71].

Instead, among the metabolites that were significantly different between good and poor simvastatin responders at baseline and could be predictive of LDL-C response, the authors reported lower predose concentrations of purine metabolite xanthine, succinic acid, stearic acid, and 2-hydroxypentanoic acid, together with higher levels of hexaric acid. All these metabolites significantly correlated with an increased responsiveness to simvastatin and, consequently, a greater LDL-C response. The purine metabolite xanthine is implicated in hydrogen peroxide production, and its lower levels in good responders are associated with a more expressed NOS signaling that catalyzes the production of NO. Indeed, xanthine oxidase has an important role as a therapeutic target for cardiovascular diseases, and xanthine oxidase inhibitors are used for the prevention of major adverse cardiovascular events [72,73]. 2-Hydroxypentanoic acid shows a reduced production in good simvastatin responders; therefore, it is very likely that simvastatin, or a metabolite of simvastatin, inhibits an enzyme that produces 2-hydroxypentanoic acid and reduces simvastatin degradation, resulting in differential pharmacokinetics. Moreover, altered levels of shikimic acid, which is an enterobacteria-derived precursor of aromatic- and indole-containing amino acids, were also observed following treatment with statins in the good responders, confirming an important influence of the gut microbiome in modulating the response to statins [64,67]. 

All these data show that simvastatin produces a more systemic effect not only focused on cholesterol metabolism and that it contributes to reducing the risk of CVDs, suggesting a critical interaction between metabolome, microbiome, and genome in the interindividual differences in response to statin therapy. The characterization of a pre-treatment metabolic signature as a panel of predictive markers can improve the classification of individuals based on the response to a particular drug, thus excluding individuals who are least likely to derive a clinical benefit. In this way, future clinical trials and pharmacological treatments will be more feasible and relevant, focusing on participant characteristics to achieve a personalizing medicine to treat individuals effectively and safely.

#### 3.1.2. Alterations in Gut Microbiota by Statin Therapy

The application of MS-based lipidomics provides a valid integrative approach to studying pharmacometabolomic changes because it gives information about whole lipid metabolism pathways that can be closely correlated to the other metabolic networks in the understanding of all mechanisms mediating statin effects [67,74]. In a recent study, the potential molecular mechanisms underlying the association between metabolic improvement and microbiota composition following simvastatin treatment were investigated to explain the gut microbiome involvement in the statin response variability [75]. A metabolomic profiling using an ultra-high-performance liquid chromatography (UHPLC) system coupled with a hybrid triple quadrupole TOF (Q-TOF) mass spectrometer was performed to study the interactions of endogenous serum metabolites with the gut microbiota following simvastatin treatment in high-lipid diet-induced hyperlipidemic rats. Differential endogenous metabolites were identified that affected the metabolism of amino acids (phenylalanine and tyrosine), unsaturated fatty acids (linoleic acid and 9-hydroxyoctadecadienoic acid), and the functions of gut microbial metabolism (*m*-coumaric acid and 3-(2-hydroxyphenyl) propionic acid) [75]. These data suggested that simvastatin therapy strongly modulates the serum metabolic profile in hyperlipidemic rats, and, since these metabolic pathways are involved in gut flora interactions, they could be potential therapeutic targets for the improvement of simvastatin hypolipidemic efficacy. Indeed, hyperlipidemia is a metabolic syndrome that is commonly linked to cardiovascular diseases. Phenylalanine is a nutrient precursor for gut microbiota-generated metabolites, which are known to be associated with cardiovascular diseases and adverse cardiovascular events, while tyrosine promotes lipid metabolism, therefore representing a potential biomarker for hyperlipidemia [75]. Both phenylalanine and tyrosine showed increased levels following statin treatment. In the same way, levels of both linoleic acid and 9-hydroxyoctadecadienoic acid were significantly increased after simvastatin therapy, which confirms their beneficial effects against cardiovascular risk, including hyperlipidemia and hypertension [75]. The concentration of the metabolites of the gut microflora, *m*-coumaric acid and a derivative of phenylpropionic acid, were also higher in hyperlipidemic rats after statin treatment, showing their antilipogenic and cholesterol-lowering properties [75].

Another recent study focused attention on the relationship between statin treatment and gut microbiota variation evaluating, for the first time, the correlations between statin-associated gut microbiota, serum metabolomic alterations, and clinical outcomes in acute coronary syndrome (ACS) patients who had or not received chronic statin treatment [76]. No significant differences were observed in the blood lipid profiles (total cholesterol, triacylglycerol, high-density lipoprotein cholesterol, and LDL cholesterol) between the ACS-statins patients and the ACS group. Moreover, since the gut microbiota is known to affect the host metabolism by producing a wide range of small compounds, the authors performed an untargeted metabolomic analysis using a UHPLC-Q-TOF system to investigate the serum metabolome [76]. Significant changes in the serum metabolomic features associated with statins therapy were reported, and the correlation analysis of these differentially abundant metabolites with the clinical phenotypes revealed that statin-positive metabolites (e.g., fatty acyls, steroids, and steroid derivatives) tended to negatively correlate with disease severity and adverse outcome events, while statin-negative metabolites (e.g., benzene and substituted derivatives, prenol lipids, and acylcarnitines) displayed opposite trends. Therefore, the authors observed in this study that ACS patients who had received long-term and standard statin therapies tended to have a better prognosis because statin therapy was associated with the restoration of gut microbiota homeostasis and improved outcomes in patients [76]. Certainly, a targeted metabolomic analysis will be needed to accurately validate the metabolic changes identified, and a larger sample size will be required to confirm the obtained data.

#### 3.1.3. Adverse Effects of Statins

The understanding of the adverse effects associated with statin treatment is another important aspect that has attracted the attention of many experts in the field. 

Statins are highly effective and safe for most people, but they can cause minor or severe side effects that should never be neglected. Statin-related myotoxicity, for example, can range from mild muscle pain up to rhabdomyolysis, which is a serious and fatal disorder that sometimes occurs in patients following pharmacological treatment [77]. Statin-associated rhabdomyolysis risk has been reported as dose-dependent and concentration-dependent [78]. Patients on intensive statin therapy must be monitored closely for muscle-related adverse effects because it is important to have an individualized statin therapy approach. The detection of myotoxicity at an early phase is essential for risk evaluation in clinical practice and several studies on rats and humans have been conducted for monitoring statin-induced skeletal muscle toxicity. Therefore, biomarkers with high sensitivity and specificity are required for early muscle injury detection. 

Metabolomics provides an accurate signature of all metabolite changes in biological fluids, cells, and tissues that can be a source for biomarker discovery. A metabolomic analysis of skeletal muscle and plasma using LC-MS and GC-MS was performed on a rat model treated with two myotoxicants, cerivastatin and tetramethyl-p-phenylenediamine, to induce a skeletal injury and identify candidate biomarkers for skeletal muscle toxicity [79]. They observed in skeletal muscle a significant increase in 2-hydroxyglutarate in cerivastatin-treated rats and hexanoylcarnitine in both types of treated rats. These increases were also measured in plasma samples at different times after dosing, demonstrating the possibility to use plasma 2-hydroxyglutarate and hexanoylcarnitine as valid and easily detectable biomarkers for the early detection of skeletal muscle toxicity in rats, with better sensitivity than the conventional markers creatine kinase and aspartate aminotransferase whose utility in clinics is limited due to their low diagnostic power [80]. Moreover, this study confirmed the importance and benefit of metabolomics for biomarker discovery in toxicological studies. Since plasma levels of acylcarnitines are linked to impaired fatty acid oxidation in skeletal muscle [81,82], it is reasonable to think that increased levels of hexanoylcarnitine observed in this study were due to altered β-oxidation activity, also supported by higher muscular levels of 3-hydroxybutyrate that is generally obtained from acetyl-CoA conversion. In addition, the mitochondrial oxidation of tetramethyl-p-phenylenediamine is crucial for its myotoxic action because muscle is rich in mitochondria and dependent on aerobic metabolism; thus, an increase in 2-hydroxyglutarate likely caused by mitochondrial dysfunction contributes to muscular dysfunction and causes redox stress [79]. More than ten years ago, based on global and targeted metabolomic profiling, Kumar et al. performed a study to discover safety urinary biomarkers for the adverse effects of atorvastatin in hyperlipidemic rats [83]. Atorvastatin is widely used and well-tolerated, but it can increase some toxicity factors such as hepatic enzymes. In this study, the authors reported liver toxicity in hyperlipidemic rats after 7 days of atorvastatin treatment based on serum biochemical parameters and histopathological tests of the liver tissue. Global metabolic profiling was obtained using LC-TOF-MS with multivariate data analysis, and several candidate biomarkers that included various steroids and amino acids were then validated by targeted metabolic profiling using GC-MS and CE-MS [83]. The authors measured low molecular weight metabolites in urine that are simple and non-invasively collected samples, and suggested estrone, cortisone, proline, cystine, 3-ureidopropionic acid, and histidine as potential biomarkers of the atorvastatin-induced hepatotoxicity. To evaluate and confirm their feasibility as potential safety biomarkers, these molecules were also quantified by GC-MS for urinary steroids and by CE-MS for amino acids. In particular, the urinary levels of proline, 3-ureidopropionic acid, and histidine increased significantly in a dose-dependent manner following atorvastatin treatment. Although it was a pilot study, these data contributed to additional insights into the role of metabolic alterations in liver toxicity of atorvastatin [83].

Among the potential statin-related adverse events, there is also an increased incidence of type II diabetes mellitus that can lead to premature discontinuation of treatment. Therefore, it is important to evaluate a correlation between statin-induced metabolic changes and statin-induced hyperglycemia and insulin resistance, to identify pre-drug treatment metabolites predictive of increased diabetic risk [84]. In this regard, a pharmacometabolomic study was performed by GC-TOF-MS on plasma pre- and post-treatment with simvastatin for 6 weeks from patients enrolled for the CAP study [84] to measure changes in intermediary metabolism and the associated high plasma glucose levels as a potentially adverse response to simvastatin. Some patients developed hyperglycemia and pre-diabetes, as well as a dysfunction of beta cells and insulin resistance in more than 50% of patients following statin therapy. An initial metabolic profile of simvastatin-induced insulin resistance was identified, including ethanolamine, hydroxylamine, hydroxycarbamate, and isoleucine, which can be predictive biomarkers of individuals at risk of developing a statin-induced new onset pre-type II diabetes mellitus [84]. In particular, the metabolite ethanolamine was identified as the most likely to predict simvastatin-induced diabetic risk, indicating that decarboxylation and oxidation were significantly associated with statin-induced hyperglycemia and insulin resistance. Therefore, this study demonstrates that the oral administration of simvastatin for 6 weeks increased the risk of developing elevated plasma glucose mostly in susceptible individuals [84]. Pharmacometabolomics allows having a baseline metabolic signature before starting the drug therapy that can be then used to find predictive biomarkers able to stratify patients and to identify subjects who are at higher risk of adverse side effects, enabling personalized selection of the most appropriate medication for each patient and personalized monitoring of their prognosis.

Another study highlights the importance of having patient-specific metabolomic and lipidomic profiles that can be used as valid predictive markers for the understanding of the adverse effects associated with statin treatment [85]. Lee et al. performed an analysis combining metabolomics and lipidomics in hyperlipidemic patients after rosuvastatin administration for 3–8 weeks [85]. Plasma and urine metabolic profiles between healthy subjects and patients with hyperlipidemia were compared to evaluate the metabolic changes following drug administration. A non-targeted global metabolomic and lipidomic analysis was performed in plasma and urine samples and led to the identification of 73 and 87 metabolites in healthy subjects and hyperlipidemia subjects, respectively. Among the identified metabolites, several molecules were found to be significantly altered between controls and patients, and they were absolutely quantified through a targeted analysis. The authors also successfully quantified 188 metabolites using a targeted approach by MRM with a UHPLC-triple quadrupole mass spectrometer, including amino acids, biogenic amines, glycerophospholipids, and sphingolipids [85]. In particular, they observed significantly decreased levels of L-carnitine, diacylglycerol, and acylcarnitines after rosuvastatin administration both in controls and patients, suggesting the lowering effect of rosuvastatin on the level of carnitines with a consequent reduction in the accumulation of acylcarnitine into the mitochondria that is important for the synthesis of fatty acyl-CoA involved in β-oxidation. Increased levels of fatty acids and lysophosphatidylcholines were only ever detected in patients with rosuvastatin, where instead a decrease in the levels of phosphatidylcholines was measured. In addition, the production of polyunsaturated fatty acids such as arachidonic acid and linoleic acid significantly increased only in hyperlipidemic patients, suggesting a decrease in β-oxidation and the consequent lower synthesis of acetyl-CoA, which could generate a mitochondrial homeostasis failure [85]. The upregulation of myristate and palmitate and the alteration of amino acids in hyperlipidemic patients after rosuvastatin treatment may also lead to a statin-induced mitochondrial dysfunction, and this could be a possible reason for the specific mild myopathy in patients. 

Due to the large use of statins, their potential impact on metabolome has also been considered in big metabolomic studies, such as a large collaborative study performed by Sliz et al. [86]. They investigated the associations between circulating metabolites and multiple lipid measures with white matter hyperintensities volume, which is an index of small vessel disease and a risk factor for all-cause mortality. Indeed, the authors stratified the population on the basis of statin treatment, considering that statin-treated individuals had the highest volume and lowest cholesterol levels.

#### 3.1.4. Beneficial Effects of Statins

Although the possible side effects of therapy with statins are unpleasant, it is important not to forget the considerable benefits of taking them for the treatment of several pathologies. Metabonomics is used not only for clinical diagnosis, but also for evaluating the clinical course of a disease, prognosis, and treatment effect of drugs, such as statins [87]. 

Many years ago, Ooga et al. performed [88] a metabolic analysis in Watanabe heritable hyperlipidemic (WHHL) rabbits as a model of hypercholesterolemia to obtain a determination of all metabolite concentrations and a characterization of the metabolic imbalance of their pathological condition. Numerous metabolites were measured in plasma and several tissues from WHHL and healthy control rabbits using CE-TOF-MS and LC-TOF-MS systems. Several significant metabolic differences between the healthy and the pathological conditions were observed, and the metabolomic features observed in the pathological rabbit model including the modulation of glutathione and phosphatidylcholine metabolism showing advanced oxidative stress in several tissues, especially in the liver [88]. In addition, the extensive reduction in the levels of purine metabolites associated with an accumulation of uric acid suggested the enzymatic activation of xanthine oxidase. The authors also evaluated changes in the metabolomic profile induced by short-term simvastatin administration and they demonstrated significant pathophysiological alterations in a portion of tissue metabolome suggestive of restoration to the healthy condition [88]. These metabolic changes were most likely due to the pleiotropic effects of statin treatment, including antioxidant action. Although these results were only an initial overview of the metabolic anatomy and further studies are required, this study showed the applicability of metabolomics for non-targeted screening to explain several aspects of hypercholesterolemia.

Shifting the attention to human subjects with hypercholesterolemia, comprehensive cross-sectional profiling of lipids and metabolites was performed by Christensen et al. in children with and without familial hypercholesterolemia (FH) aiming to characterize the alterations associated with elevated LDL-C in FH patients [89]. Elevated plasma cholesterol is the most important risk factor for atherosclerosis and cholesterol-lowering treatment with statins is required to stop or slow down atherosclerotic development in FH children. Plasma metabolites were measured by high-throughput NMR spectroscopy to compare the differences between statin-treated and non-statin-treated FH children, and healthy children [89]. It is important to investigate hypercholesterolemia-associated metabolic aberrations in HF children to better understand the disease, and thereby improve the treatment of hypercholesterolemia in children and, more in general terms, the treatment of atherosclerotic processes. The authors observed increased levels of atherogenic ApoB-containing lipoproteins and lipid fractions in both statin-treated and non-statin-treated FH children compared to healthy children. In addition, FH children showed alterations in HDL subfractions, and in particular, their small HDL particles were characterized by a higher content of cholesteryl esters, and lower levels of free cholesterol and phospholipids [89]. Therefore, these metabolic changes caused by hypercholesterolemia suggested an impaired reverse cholesterol transport system. Increased levels of plasma fatty acids, such as polyunsaturated fatty acids (PUFAs) and linoleic acid, were also reported in non-statin-treated hypercholesterolemic children, whereas acetoacetate and acetate were lower compared with healthy children. No significant differences were then detected in beta-hydroxybutyrate, glycoprotein, creatinine, albumin, or any glucose or amino acid metabolites between the groups. 

In summary, the authors demonstrated that many lipid-related and metabolic species were positively or negatively associated with hypercholesterolemia in FH children and healthy children, and statin-treated FH children showed a pattern of lipid-related and metabolic signatures closer to healthy children, compared with non-statin-treated FH children, confirming the beneficial effect of statins as a treatment for hypercholesterolemia [89]. Of course, this should be considered a pilot study, and the need to extend the number of participants is evident to confirm the observed differences, but the present study showed how NMR-based metabolomics and lipidomics have the potential to become future powerful tools for risk stratification and treatment evaluation in children with hypercholesterolemia.

It is well known that statins cause not only lipid reduction but also several pleiotropic effects, such as antioxidant properties, anti-inflammatory effects, and immunomodulation [90]. In addition, statins show antimicrobial activity, as has been shown in several studies [91,92,93]. In a recent study, for the first time, the antimicrobial effect of simvastatin was evaluated on proteome and metabolome in Escherichia coli by LC-MS-based label-free proteomic analysis and GC-MS-based metabolomics [94]. Differentially expressed proteins and metabolites between control and simvastatin-treated groups were measured to find altered molecules under the simvastatin stress condition. The authors demonstrated that simvastatin treatment affects both metabolome and proteome structures. In particular, the biosynthesis of amino acids was changed under statin stress, tricarboxylic acid cycle and glyoxylate shunt were altered, and pyruvate metabolism was downregulated in the simvastatin-treated group. Moreover, various metabolites in purine and pyrimidine metabolism were altered by the effect of simvastatin. Therefore, simvastatin caused increased reactive oxygen species (ROS) production and a significant change in the energy metabolism of Escherichia coli to adapt to stress conditions. These integrated metabolomics and proteomics data allowed a global evaluation of the simvastatin effects on the phenotype of Escherichia coli suggesting some important antimicrobial targets and cellular pathways involved in the response to stress conditions, thus providing more details in the comprehension of the mechanisms of action for simvastatin on various biological pathways [94]. This paper is an example of the use of metabolomics for drug repurposing, also known as drug repositioning, which is an efficient strategy for identifying new pharmacological activities or therapeutic purposes for already-approved or investigational drugs that are different from the original medical indications [95,96]. Metabolomics, together with advanced bioinformatic tools and pathways analysis, has successfully contributed to improving drug repurposing for identifying new uses for existing drugs through the generation of large-scale metabolic databases mapping molecular alterations under a specific drug treatment in disease conditions [16]. Indeed, the repositioning of statins has been also suggested for age-related macular degeneration, which is characterized by altered lipid homeostasis, even if a conclusive result is still lacking [97]. The beneficial systemic effects of the use of statin therapy on many lipids and other circulating metabolic biomarkers of cardiovascular risk were also investigated by conducting a metabolomic profiling at two-time points in four population-based cohorts using a high-throughput NMR platform [98]. In this multicenter study, the concentration changes in the serum or plasma levels of several lipids and metabolites during follow-up were compared between subjects who started statin therapy and persistent non-users. In addition, to confirm that the observed lipoprotein, fatty acid, and metabolite alterations were due to the effects of statins, the authors applied Mendelian randomization by using a genetic variant in the *HMG-CoA reductase* (*HMGCR*) gene, known to affect hepatic HMGCR expression and circulating LDL-C, as a proxy for the pharmacological action of statins [98]. Thanks to this *HMGCR* gene variant mimicking the effect of statins, the authors were able to compare the genetic association pattern to the metabolic changes observed. Starting statin therapy was associated with several lipoprotein and fatty acid variations consistent with their cardioprotective effects, among which a pronounced decrease in remnant cholesterol in a similar extent as ApoB (80% relative to the LDL-C-lowering effect) and a modest lowering of VLDL and total triglycerides. Omega-6 fatty acids, such as linoleic acid, showed the most evident reduction associated with the use of statin. Instead, no significant changes were measured for circulating amino acids, ketones, and glycolysis- and gluconeogenesis-related metabolites both at the metabolic and genetic levels, suggesting minimal pleiotropic effects of statin use on these non-lipid biomarkers [98]. This study highlighted a close match between the metabolic changes associated with statin use and the genetic association pattern with the variant in the *HMGCR* gene, demonstrating that the observed metabolic modulations were a consequence of the mechanism-based effect of statins. These findings confirmed how metabolomics in combination with genetic proxies for drug mechanisms can explain the molecular effects of known targets, evaluate the pharmacological mechanisms, and suggest an appropriate drug therapy or novel drug targets [98].

Another common application of metabonomics is the study of the development and progression of diseases following a specific pharmacological treatment.

Luo et al. applied metabonomics to study the influence of atorvastatin on the metabolic pattern of rats with pulmonary arterial hypertension (PAH) [99]. NMR was used to detect and analyze the serum metabolites and, thus the relationship between metabolic changes and pulmonary artery remodeling. This study aimed to better understand the changes in pulmonary artery remodeling and pulmonary arterial pressure in rats with PAH at different time points after atorvastatin treatment, and the authors found differential serum metabolites able to distinguish the groups of patients [99]. Moreover, a significant increase in carnitine was observed only in the group of patients treated with atorvastatin for one week, indicating that the β-oxidation of fatty acid was significantly inhibited by the drug. This was also confirmed by the inhibition of the Warburg effect [100] which was not observed after the second week of treatment. A significant alteration of the levels of glycogen synthase kinase-3β (GSK-3β), hexokinase 2 (HK-2), sterol regulatory element-binding protein 1c (SREBP-1c), and carnitine palmitoyltransferase I (CPT-1) was also observed in the lung tissues [99]. In particular, GSK-3β and SREBP-1c were decreased, whereas HK-2 and CPT-1 were increased in PAH patients. Therefore, the results indicated that atorvastatin significantly improved pulmonary artery remodeling and reduced pulmonary artery pressure. This study provides novel information on the potential mechanisms involved in PAH development and progression, as well as evidence of the beneficial effects of statin treatment in patients.

### 3.2. PCSK9 Inhibitors

PCSK9 inhibitors are pharmacological agents used to reduce blood LDL-C levels and improve cardiovascular outcomes both in primary and secondary prevention [101]. They are human monoclonal antibodies that bind PCSK9 protein with high affinity to lower LDL-C concentrations by blocking the degradation of cholesterol receptors available on the hepatocyte cell surface, which are responsible for removing LDL-C from blood [102]. PCSK9 inhibitors seem to show a more effective lipid-lowering profile than statins [103], even if the efficacy and safety among PCSK9 inhibitors and statins are still a subject of intensive study. Recently, a network meta-analysis performed by Zhao et al. reported that pharmacological treatments with statins and PCSK9 inhibitors offered a nearly identical decrease in cardiovascular events in patients with hypercholesterolemia, but PCSK9 inhibitors were the most effective agent in improving lipid levels and not associated with any increased risk of statin-related side-effects [101]. Therefore, PCSK9 inhibitors can be considered as an alternative lipid-lowering therapy for patients with hypercholesterolemia, especially for those with statin intolerance or resistance. 

A randomized, double-blind, placebo-controlled trial, known as FOURIER study, involved about 27,000 patients with atherosclerotic cardiovascular disease and high LDL cholesterol levels (a median of 92 mg per deciliter at baseline) who received statin therapy and were randomly assigned to receive also evolocumab or placebo as subcutaneous injections [104]. Evolocumab is a PCSK9 inhibitor that, in combination with a statin, reduced LDL-C levels by approximately 60% in comparison to a placebo, with a median of 30 mg per deciliter that was maintained over time. Moreover, evolocumab significantly reduced related atherogenic lipid measures, such as the levels of non-HDL cholesterol by 52% in comparison with a placebo, and ApoB concentration by 49%. Moreover, this trial had a relatively short duration of follow-up because it was stopped after only 2.2 years of follow-up; thus, it would be advisable to evaluate both the longer-term benefits and longer-term safety to extend and confirm the evidence that emerged in this study [104].

A Mendelian randomization study by Ference et al. also compared the effects of lower LDL-C levels mediated by variants located in *HMG-CoA reductase* (*HMGCR*), the gene encoding the target of statins, or in *PCSK9* on the risk of cardiovascular events and the risk of diabetes [105]. The results showed that variants in *PCSK9* had a nearly identical effect as statin therapy on the risk of cardiovascular diseases and diabetes per unit decrease in plasma LDL-C level. Of note, the clinical benefit of *PCSK9* and *HMGCR* variants increased when presented together. 

Based on evidence from the two major clinical trials on PCSK9 inhibitors, the FOURIER [104] and the ODYSSEY [106] outcome trials that used evolocumab and alirocumab, respectively, as fully humanized monoclonal antibodies against PCSK9, a recent paper by Gallego-Colon et al. underlines that the 2019 European Society of Cardiology/European Atherosclerosis Society guidelines for the management of dyslipidemias establish the use of PCSK9 inhibitors to very high-risk atherosclerotic cardiovascular disease patients who are unresponsive to a maximum tolerated dose of statins and ezetimibe [107]. Therefore, the discovery of PCSK9 inhibitors has defined a new era of lipid-lowering therapies for patients with atherosclerotic cardiovascular disease which can change future clinical practice.

Sliz et al. evaluated the potential differences between metabolic effects of PCSK9 inhibitors and statins, performing the lipid and metabolite profiling of a large randomized statin trial and comparing the obtained results with the effects of genetic inhibition of PCSK9 in large population studies, acting as a naturally occurring trial of PCSK9 inhibitors [108]. The authors quantified about 200 circulating lipids and metabolites by high-throughput NMR, including lipoprotein subclasses, their lipid concentrations and composition, fatty acids, and amino acids, in 5359 blood samples from the PROSPER (PROspective Study of Pravastatin in the Elderly at Risk) trial at 6 months post-randomization [109], and in more than 70,000 samples from eight population cohorts using *PCSK9* rs11591147 as a loss-of-function mutation that mimics the therapeutic effects of PCSK9 inhibitors (i.e., lower LDL-C levels and reduced cardiovascular risk). PROSPER is a double-blind, randomized placebo-controlled study investigating the benefit of pravastatin in elderly individuals with a history of, or risk factors for, cardiovascular disease and stroke [109]. The effects of genetic inhibition of PCSK9 and statin treatment on the measured metabolic markers were comparable [108]. *PCSK9* rs11591147 also displayed similar effects as statin therapy for alterations in lipoprotein lipid composition and fatty acid distribution. However, some differences were observed for VLDL lipids, and genetic inhibition of PCSK9 had a weaker effect on lowering VLDL-C compared with statins for an equivalent lowering of LDL-C [108]. This could potentially contribute to smaller reductions in cardiovascular disease risk. Genetic inhibition of PCSK9 showed no significant effects on amino acids, glycolysis-related metabolites, ketone bodies, and the inflammation marker glycoprotein acetylation (GlycA), whereas statin treatment caused minor effects on these metabolites and weakly lowered GlycA and isoleucine levels [108]. Therefore, this study highlights the importance of large-scale metabolomic profiling in combination with genetics and randomized trials to evaluate the differences in many circulating metabolic biomarkers and to clarify the potential therapeutic differences in the molecular mechanisms to reduce cardiovascular risk.

A recent study by Zhang et al. showed that the inhibition of PCSK9 with evolocumab significantly reduced VLDL particle concentrations depending on the baseline lipoprotein(a) (Lp(a)) level, as well as lowering LDL-C [110]. Indeed, the authors demonstrated an important effect of evolocumab on VLDL metabolism in subjects with elevated Lp(a), which is known to have an increased cardiovascular risk. The extent of VLDL reduction was dependent on the baseline Lp(a) level, thus subjects with higher baseline Lp(a) showed a tendency to have a larger reduction in VLDL concentration. Detailed NMR metabolomics was performed on plasma samples from subjects with elevated Lp(a), which were randomly divided into two groups treated with placebo and evolocumab, at baseline and 16 weeks after treatment [110]. Quantification of 225 metabolic measures was performed focusing on multiple metabolic pathways including lipoprotein subclasses, fatty acids, amino acids, and glycolysis intermediates. Evolocumab treatment caused a 17% reduction of circulating Lp(a), together with a significant decrease in VLDL, intermediate-density lipoprotein (IDL), and LDL particles, and their lipid contents. Moreover, the inhibition of PCSK9 was associated with a 30% reduction of total fatty acids, in particular docosahexaenoic acid levels. No differences were observed in concentrations of other circulating metabolites such as amino acids, glycolysis, and ketone bodies [110]. Certainly, further studies are required to verify if evolocumab has a similar effect on VLDL in individuals without elevated Lp(a) concentration.

An untargeted metabolomics approach was also performed to obtain a global view of metabolic and lipidomic pathways and characterize metabolites and lipids that were modified in plasma from patients with FH who received treatment with PCSK9 inhibitors [111]. Familial hypercholesterolemia causes extremely high circulating LDL-C levels, which are due to mutations of different genes involved in LDL-C metabolism, such as *PCSK9*. After 12 weeks of treatment with evolocumab, the authors observed a significant reduction of LDL-C levels compared to baseline, together with increments in creatine, indole, and indoleacrylic acid concentrations. Instead, a significant decrease in choline and phosphatidylcholine levels, as well as a reduction in platelet-activating factor 16, were reported. This study highlighted for the first time a reduction in inflammation and platelet activation metabolites in FH patients after therapy with PCSK9 inhibitors [111]. Moreover, due to the small sample size, further studies are required to clarify the underlying mechanisms and the impact on cardiovascular events, confirming data in a larger number of participants with targeted analysis.

In the same FH patients examined in this study [111], the authors have already shown a decrease in small dense LDL, known to be more susceptible to oxidative modification and widely recognized as predictors of atherosclerosis, coronary heart disease, and stroke, suggesting an association between their reduction and changes in oxidation markers and endothelial function in FH patients receiving PCSK-9 inhibitors [112]. Indeed, they reported a significant decrease in the urinary excretion of 11-dehydro-thromboxane, a major index of in vivo platelet activation, and 8-isoprostaglandin-2alpha, which is a biomarker of lipid peroxidation, following a 12-week treatment with evolocumab. In this study, they also noticed a significant reduction of Lp(a) levels after therapy, and Lp(a) is known to be a highly atherogenic lipoprotein [112]. Therefore, besides the evolocumab-related lipid-lowering effect in FH patients, the treatment improved endothelial function which is a documented predictor of cardiovascular events. 

### 3.3. Fibrates

Fibrates are activators of peroxisome proliferator-activated receptor alpha (PPARα), used to prevent and treat hyperlipidemia often in combination with statins, thanks to their ability to increase fatty acid β-oxidation, fatty acid transport, and HDL metabolism, leading to a global reduction of triglyceride and cholesterol levels [50,113].

Patterson et al. identified pantothenic acid and acylcarnitines as specific potential indicators of PPARα activation of fatty acid β-oxidation induced by fibrates using a metabolomic approach [114]. They treated healthy volunteers with fenofibrate (200 mg/day) for 14 days and analyzed urinary metabolites at time 0, after 2 days, and after 14 days, by LC-MS, using an ultra-performance liquid chromatography (UPLC) system coupled to a high-resolution mass spectrometer (Q-TOF). They evidenced a dramatic decrease in urinary pantothenic acid (>5 fold) and acylcarnitines (>20 folds), and to confirm that these molecules could be biomarkers of PPARα activation, they treated wild-type and Ppara-null mice with 0.1% fenofibrate for 7 days. Of note, only wild-type mice exhibited a reduction of both urinary pantothenic acid (40 folds) and acylcarnitines (88 folds), suggesting that the effect is strongly associated with the activation of PPARα and transcends species [114]. Considering that it has also been demonstrated that enzymes involved in the metabolism of pantothenic acid (i.e., pantothenate kinase) and short-chain acetyl carnitine (i.e., carnitine palmitoyltransferase) are upregulated by PPARα activation to increase beta oxidation in mitochondria, their reduction in the urine can be justified and can be considered a more specific marker of fibrate activity with respect to cholesterol levels. Despite being performed on 10 healthy volunteers, this study highlights the biological variability of metabolic response in different subjects due to genetic or environmental (i.e., diet) differences because abnormal behaviors were shown in two of them.

Further, a combined transcriptomic and metabolomic approach has been applied to compare in a mouse model 2 weeks of fenofibrate treatment with respect to fish oil treatment [115]. Fish oil is indeed rich in eicosapentaenoic acid and docosahexaenoic acid, fatty acids that act through PPARα activation and suppress the activity of the prolipogenic transcription factor SREBP-1. Fish oil specifically decreased the levels of various phospholipid species, while fenofibrate specifically increased the levels of Krebs cycle intermediates (i.e., fumaric acid, isocitric acid, malic acid, succinic acid and α-ketoglutaric acid) and most amino acids. These data correlate well with the induction of genes involved in the Krebs cycle and in the urea cycle or in the metabolism of amino groups. Comparing both plasma metabolome and hepatic transcriptome, it emerged that despite being similarly potent toward modulating plasma lipids, fish oil caused only modest changes in gene expression likely in comparison to fenofibrate, reflecting the activation of multiple mechanistic pathways with fish oil, typical of nutritional interventions [115].

Despite the established protective effect of fibrates and their general safe use in humans, there is some evidence in rodents of peroxisome proliferation and hepatocarcinogenesis, whose mechanism is not completely understood. Ohta et al. applied an untargeted metabolomic approach, using GC-MS and LC-MS, as a tool to evaluate the toxicology of fenofibrate in rats treated with fenofibrate 300 mg/Kg/day or vehicle for a total of 14 days, and they compared both plasma and urinary metabolites at two time points (2 days and 14 days) [113]. In addition, confirming the canonical effects of fibrates, such as the reduction of β-oxidation with the reduction of urinary levels of carnitine and the increase in plasma 3-hydroxybutanoic acid, they identified novel metabolic changes. Indeed, they evidenced the alteration of a panel of metabolites involved in liver dysfunction (i.e., bile acids increase) and renal dysfunction (1-methylguanidine), suggesting them as potential biomarkers of fibrate toxicity. Regarding the issue of carcinogenesis, the authors focus their attention on the perturbation of glutathione biosynthesis and oxidative stress [113]. Of note, in their conditions they demonstrated a reduction of tricarboxylic acid cycle intermediates, opposite to the effects shown in mice by Lu et al. [115].

#### Combination Therapy of Statins and Fibrates

Fibrates are frequently used in combination with statins, working in synergy to reduce plasma lipids, even if this type of treatment is associated with a higher incidence of fatal side effects, such as acute tubular necrosis and rhabdomyolysis. Several hypotheses have been formulated including pharmacokinetic interference, displacement of statins from their binding sites, synergistic action on skeletal muscle, or inhibition of statin glucuronidation by fibrates [116]. Strauss et al. used a metabolomic tool to disentangle this issue in healthy rats treated for 4 weeks with monotherapy or a binary combination of fibrates (fenofibrate or clofibrate) and statins (atorvastatin or pravastatin). They integrated targeted GC-MS and LC-MS to analyze plasma polar and non-polar metabolites at different time points. No drug accumulation was shown, suggesting the absence of pharmacokinetic interferences. In general, combination therapy led to under-additive effects. Indeed, many metabolites were also modulated with monotherapy, but they identified additional metabolites modulated by combined therapy, which are related to the control of vascular tone and antioxidant status, such as higher levels of 5-oxoproline, glutamine, and glycine involved in glutathione activity and synthesis, which anticipate an altered redox status that might affect muscle, or lower levels of tryptophan, the precursor of serotonin, a known vasoconstrictor [116]. Of note, when analyzing the effects of monotherapies, the authors confirmed the effect of fenofibrate on kynurenic acid, pantothenic acid and glycine observed by Ohta et al. at urine levels [113].

While Strauss et al. designed their study in healthy rats to evidence mainly the side effects of combined therapy [116], Xu et al. performed a GC-MS-based metabolomic study of plasma in a diet-induced hyperlipidemia rat model treated with simvastatin or fenofibrate in monotherapy [50]. They successfully identified many potential targets changed by hyperlipidemia and normalized by both drugs, such as cholesterol, beta-hydroxybutyric acid, linoleic acid, creatinine, and ornithine. Of note, the reduction of plasma levels of tyrosine was recovered only by fenofibrate.

More recently, Han et al. evaluated in human subjects with combined hyperlipidemia the effects of atorvastatin escalation with atorvastatin/fenofibrate combination [117]. Serum metabolite profiling revealed distinct metabolite clustering with the combined therapy with respect to statin escalation, including the reduction of acylglycerols and many ceramides, and the increase in serum levels of sphingomyelins and L-carnitine. On the other hand, only atorvastatin escalation decreased lysophosphatidylcholines [117].

### 3.4. Nutraceutical and Dietary Habits

In the past decades, an increasing number of studies have suggested that nutraceuticals and dietary habits may be also effective for CVD prevention [118,119,120], with significant effects on reducing CVD risk and population mortality [121].

In particular, natural micronutrients and non-nutrient components in these foods, such as polyphenols, have been shown to modulate cholesterol metabolism [121]. Sommella et al. focused their attention on *Malus pumila* Miller cv. Annurca, an apple native to southern Italy, containing high levels of procyanidin B2, a dimeric procyanidin, with favorable biochemical effects against metabolic disorders and atherosclerosis [121]. They demonstrated that 800 mg/day of Annurca apple polyphenolic extract (AAE) substantially reduced both LDL-C (37.6%) and increased HDL-C (49.3%), similarly to statin treatment [122], and applied an untargeted metabolomic approach to depicting the molecular mechanism activated by this nutraceutical treatment [121]. They used deuterium labeling for 72 h coupled with GC-MS and Fourier transform-ion cyclotron resonance (FT-ICR) mass spectrometry to highlight primary metabolic pathways influenced by AAE in in vitro cultured human hepatocytes, HuH7 cells. Their results suggested that AAE acts differently from statins, promoting mitochondrial activity, reprogramming fatty acid metabolism, and inhibiting lipogenesis and cholesterogenesis. AAE diverts acetyl-CoA to the Krebs cycle to produce adenosine triphosphate (ATP) and energy for the cell, instead of becoming HMG-CoA. Glutamine levels are also reduced by AAE suggesting that glutamine can be indeed one of the sources of increased mitochondrial activity. Furthermore, AAE stimulates glycolysis ultimately increasing mitochondrial respiration. Thus, inhibition of lipogenesis and cholesterogenesis could be ascribed to a modulation of the entire metabolic process connected with the use of citrate. Of note, no deuterium incorporation could be measured in any of the fatty acids over-represented in AAE treated cells, indicating that the apple polyphenols induce their release from intracellular lipid stores, probably triglycerides (TGs) and plasma membrane lipids, instead of increasing de novo synthesis [121].

A serum metabolomic study was performed by Xu et al. using an LC-MS-based approach to study the effects of 45 days of oat supplementation on serum lipids in adults with mild hypercholesterolemia and to understand the underlining mechanisms [123]. They showed the reduction of total cholesterol (TC) (−8.41%) and LDL-C (−12.9%) and identified 7 upregulated metabolites and 14 reduced metabolites, suggesting regulation of glycerophospholipid, alanine, aspartate, glutamate, sphingolipid, and retinol metabolism [123]. The negative association between CVD and whole-grain consumption has been widely demonstrated and ascribed to the ability of the functional compound beta-glucan to prevent the absorption of cholesterol in the intestine, but metabolomic studies revealed alteration of fatty acid biosynthesis, amino acid biosynthesis, and metabolism [124,125,126].

In addition, the beneficial effects of probiotics to improve lipid profiles have been demonstrated in animal models and humans. Ding et al. studied the effects of *Lactobacillus plantarum* LP3, from traditional fermented yak milk, on the plasma lipid profile, gut microbiota, and cecum metabolome, by LC-MS, in rats treated with a high-fat diet [127]. Together with a significant reduction of TC, TG, and LDL-C, they evidenced adjustments in the biosynthesis of fatty acids, steroids, and bile acids, and the metabolism of linoleic acid, linolenic acid, and arachidonic acid were the main metabolic pathways in obese rats. The ability of *Lactobacillus plantarum* LP3 to reduce the ratio of Firmicutes to Bacteroidetes in obese rats could explain the reduction in metabolites associated with the biosynthesis of fatty acids [127].

Dietary plant-derived polyphenols are another class of molecules with protective effects against cardiovascular diseases [128]. Zhou et al. used a metabolomic approach based on GC-MS analysis of extracts from liver tissues to evaluate the synergistic protective effects of quercetin and resveratrol in mice that were fed a high-fat diet [129]. The integration of metabolomic and transcriptomic results clearly showed the enhancement of glycolysis, fatty acid oxidation, and gluconeogenesis. The metabolites that were reduced due to the high-fat diet resulted in being restored by quercetin or resveratrol treatment, such as 4-aminobutyric acid, ornithine, histidine, and lysine [129].

## 4. Conclusions

In this review, we reported the most relevant pharmacometabolomic studies investigating the effects of lipid-lowering therapies with high-throughput approaches, highlighting the main biomarkers or pathways alterations due to pharmacological treatment (Table 1). Regrettably, the number of lipid-lowering drugs analyzed from a metabolomic point of view is limited in respect to the complete panel of treatments that are now available, suggesting that pharmacometabolomic studies are still in their infancy in this field. 

However, it is increasingly evident that metabolomic approaches in pharmacology could be useful not only in the understanding of drug safety, toxicity, and metabolism, but also in the prediction of drug response and in the identification of biological mechanisms, even if some limitations should be acknowledged. Indeed, there is still a lack of standardized protocols for both sample preparation (i.e., collection, storage, and processing) and data acquisition, which are very important for a clinical application of these approaches. Data have been obtained in different compartments, both in clinical settings or animal models, at different times, thus making it difficult to compare them and have a comprehensive view of the metabolomic effects. Another important issue that should be taken into consideration is the influence of the environment (i.e., smoking, food, and physical activity) on the metabolic phenotype, thus requiring a large number of samples to obtain reproducible results, as well as very accurate experimental design. It would be important to also perform longitudinal studies increasing the compliance of patients with the introduction of remote sampling or less invasive collection procedures.

In conclusion, this review highlights the importance of pharmacometabolomics studies as a tool to uncover alterations of metabolic phenotype and we can envisage that the integration of “omics” approaches, including genomics, proteomics, and metabolomics, could also help in the definition of a precision medicine approach in the field of lipid-lowering therapies. Moreover, metabolomics combined with multi-omics strategies and advanced bioinformatics tools could definitely improve the drug repurposing which has gained importance in recent years for identifying novel therapeutic indications for already-registered drugs. Since lipid-lowering drugs have pleiotropic effects beyond their known mechanism of action, the discovery of repurposed drugs has implications for precision medicine to treat individual patients providing a decrease in the cost of a new drug development and benefits for the treatment of cardiovascular diseases [130].

## Figures and Tables

**Table 1:** Summary of the main metabolomic studies on the effects of lipid-lowering therapies.

Treatment	Sample Type	Inclusion Criteria	Exclusion Criteria	Matrix	Experimental Design	Analytical Technique	Type of Analysis	Main Biomarkers or Pathways Involved	Ref.
Statins									
Atorvastatin	Human healthy subjects	Selection based on medical history and routine clinical laboratory tests (i.e., hematology, urinalysis, biochemistry, serology, and physical examination).	History or indication of renal, gastrointestinal, or hepatic abnormality; Acute or chronic disease.	Plasma	Randomized open-label clinical trial	GC-MS	Untargeted	Tryptophan, alanine, arachidonic acid, 2-hydroxybutyric acid, cholesterol, and isoleucine	[12]
Simvastatin	Human healthy subjects	African American and Caucasian individuals; Age ≥ 30 years; Baseline total serum cholesterol level of 160–400 mg/dL; Selection based on demographic characteristics, medical history, risk factors for coronary heart disease, physical examination findings, and laboratory data; Six weeks on simvastatin therapy (40 mg at bedtime) Selection of participants from the entire population enrolled: “good and poor responders” from the top and bottom 10% tails of the LDL-C response distribution (response to therapy defined as the percentage change in LDL cholesterol from baseline).	Inability to maintain ≥90% compliance with the study medication; Use of lipid-lowering medication (or over-the-counter products containing sterol or stanol esters or fish oil fatty acids); Change in dietary intake or a weight change of ≥4.5 kg; Use of corticosteroids, immunosuppressive drugs, or drugs affecting the CYP3A4 system; Known liver disease or elevated transaminase levels more than twice the upper limit of normal; Elevated creatine phosphokinase levels > 10 times the upper limits of normal; Uncontrolled hypertriglyceridemia, blood pressure, or diabetes mellitus; Abnormal renal or thyroid function; Alcohol or drug abuse; Major illness in the preceding 3 months; Pregnancy; Known intolerance to statins.	Plasma	Non-randomized open-label clinical trial	GC-MS	Targeted	Arachidonic acid and linoleic acid within primarily phosphatidylcholine and cholesteryl esters, plasmalogens	[63]
Simvastatin	Human healthy subjects	African American and Caucasian individuals; Age ≥ 30 years; Baseline total serum cholesterol level of 160–400 mg/dL; Selection based on demographic characteristics, medical history, risk factors for coronary heart disease, physical examination findings, and laboratory data; 6 weeks on simvastatin therapy (40 mg at bedtime); Selection of participants from the entire population enrolled: (1) “good and poor responders” from the top and bottom 10% tails of the LDL-C response distribution (response to therapy defined as the percentage change in LDL cholesterol from baseline), (2) “full range responders” randomly selected from the entire range of LDL-C response, excluding participants who had been selected for the extreme range group.	Inability to maintain ≥90% compliance with the study medication; Use of lipid-lowering medication (or over-the-counter products containing sterol or stanol esters or fish oil fatty acids); Change in dietary intake or a weight change of ≥4.5 kg; Use of corticosteroids, immunosuppressive drugs, or drugs affecting the CYP3A4 system; Known liver disease or elevated transaminase levels more than twice the upper limit of normal; Elevated creatine phosphokinase levels >10 times the upper limits of normal; Uncontrolled hypertriglyceridemia, blood pressure, or diabetes mellitus; Abnormal renal or thyroid function; Alcohol or drug abuse; Major illness in the preceding 3 months; Pregnancy; Known intolerance to statins.	Plasma	Non-randomized open-label clinical trial	GC-MS	Targeted	Lithocholic acid, taurolithocholic acid, glycolithocholic acid, and coprostanol	[64]
Simvastatin	Human healthy subjects	African American and Caucasian individuals; Age ≥ 30 years; Baseline total serum cholesterol level of 160–400 mg/dL; Selection based on demographic characteristics, medical history, risk factors for coronary heart disease, physical examination findings, and laboratory data; 6 weeks on simvastatin therapy (40 mg at bedtime); Selection of participants from the entire population enrolled: (1) “good and poor responders” from the top and bottom 10% tails of the LDL-C response distribution (response to therapy defined as the percentage change in LDL cholesterol from baseline), (2) “full range responders” randomly selected from the entire range of LDL-C response, excluding participants who had been selected for the extreme range group.	Inability to maintain ≥90% compliance with the study medication; Use of lipid-lowering medication (or over-the-counter products containing sterol or stanol esters or fish oil fatty acids); Change in dietary intake or a weight change of ≥4.5 kg; Use of corticosteroids, immunosuppressive drugs, or drugs affecting the CYP3A4 system; Known liver disease or elevated transaminase levels more than twice the upper limit of normal; Elevated creatine phosphokinase levels >10 times the upper limits of normal; Uncontrolled hypertriglyceridemia, blood pressure, or diabetes mellitus; Abnormal renal or thyroid function; Alcohol or drug abuse; Major illness in the preceding 3 months; Pregnancy; Known intolerance to statins; Minors.	Plasma	Non-randomized open-label clinical trial	GC-MS	Untargeted	*Pre- vs. post-treatment*: lauric acid, alpha and gamma tocopherols, 2-hydroxyvaleric acid, threonine and oxalic acid; *Correlation with LDL-C response to simvastatin*: cystine, glutamine, urea cycle intermediates, ornithine, citrulline and lysine; *Good vs. poor responders*: xanthine, 2-hydroxyvaleric acid, succinic acid, stearic acid, and fructose.	[67]
Simvastatin	Hyperlipidemic rats	Sprague Dawley male rats who were fed a high-lipid diet for 4 weeks to induce hyperlipidemia; Approximately 8 weeks old; Weighing 180–220 g.		Serum	Animal study	LC-MS	Untargeted	Phenylalanine, tyrosine, linoleic acid, 9-hydroxyoctadecadienoic acid (9-HODE), *m*-coumaric acid, and 3-(2-hydroxyphenyl) propionic acid	[75]
Long-term and standard statins (not mentioned the type of statin)	Human healthy subjects and acute coronary syndrome patients	Male or female patients who exhibited ≥50% stenosis in at least one main coronary artery based on coronary angiography images; The main admitting diagnosis of patients was unstable angina, NSTEMI or STEMI; Healthy volunteers who exhibited no CAD-related clinical symptoms or signs or exhibited negative results upon coronary artery CT or coronary angiography, and who had not taken any statins within 4 weeks before recruitment.	Antibiotic treatment for more than three consecutive days within 3 months before enrollment; Gastrointestinal diseases or received gastrointestinal surgery within 1 year; Any malignant tumors, autoimmune disorders, infectious diseases or severe renal dysfunction (creatine >3.0 mg/dL)	Serum	Interventional study (case vs. control)	LC-MS	Untargeted	Fatty acyls, steroids, and steroid derivatives, benzene and substituted derivatives, prenol lipids, and acyl carnitines	[76]
Atorvastatin	Hyperlipidemic rats	Sprague Dawley male rats with hyperlipidemia induced by intraperitoneal injections of poloxamer-407 saline solution (1 g kg^−1^) every 3 days; Six to eight weeks old; Weighing 280–325 g.		Urine	Animal study	LC-MS, GC-MS and CE-MS	Untargeted and targeted	Estrone, cortisone, proline, cystine, 3-ureidopropionic acid, and histidine	[83]
Cerivastatin	Rat	Fischer male rats at 8 weeks of age who were fed a diet supplemented with cerivastatin or commercial diet only as a control.		Plasma and skeletal muscle tissue	Animal study	LC-MS and GC-MS	Untargeted	2-Hydroxyglutarate and hexanoylcarnitine	[79]
Simvastatin	Human healthy subjects	African American and Caucasian individuals; Age ≥ 30 years; Baseline total serum cholesterol level of 160–400 mg/dL; Selection based on demographic characteristics, medical history, risk factors for coronary heart disease, physical examination findings, and laboratory data; Six weeks on simvastatin therapy (40 mg at bedtime); Selection of participants from the entire population enrolled: 60 years of age.		Plasma	Non-randomized open-label clinical trial	GC-MS	Untargeted	Ethanolamine, hydroxylamine, hydroxycarbamate, and isoleucine	[84]
Rosuvastatin	Human healthy subjects and hyperlipidemic patients	Healthy Korean volunteers in the age range of 20–50 years; Subjects abstained from consuming caffeine, alcohol, and tobacco during hospitalization; Hyperlipidemic patients in the age range of 20–55 years whose LDL level was over 130 mg∙dL^−1^.	Any past medical or medication history or any abnormal findings based on a physical examination, clinical laboratory tests or vital signs; High LDL levels; Taking any lipid-lowering agent within the previous 6 weeks.	Plasma and urine	Interventional study (case vs. control)	LC-MS	Untargeted and targeted	L-carnitine, diacylglycerol, acylcarnitines, fatty acids, lysophosphatidylcholines, phosphatidylcholines, arachidonic acid, linoleic acid, myristate and palmitate	[85]
Simvastatin	Hyperlipidemic rabbits	Japanese White male rabbits and Watanabe heritable hyperlipidemic rabbits aged 11 months.		Plasma and tissues (liver, aorta, cardiac muscle, and brain)	Animal study	CE-MS and LC-MS	Untargeted	Glutathione and phosphatidylcholine metabolism, purine compounds, and uric acid	[88]
Atorvastatin, rosuvastatin and simvastatin	Children with and without familial hypercholesterolemia	Healthy control children within a normal range of plasma LDL-C (divided into normal-high and normal-low LDL-C, with respect to the median LDL-C 2.13 mmol/L); Children with a definite familial hypercholesterolemia diagnosis based on clinical or genetic diagnosis; Some familial hypercholesterolemia children were on statin treatment, while other were non-statin users at the time of blood sampling; Healthy control children and FH children were similar with respect to gender, HDL-C, triglycerides, glucose and C reactive protein.		Plasma	Cross-sectional study	NMR	Untargeted	Cholesteryl esters, free cholesterol and phospholipids in small HDL, polyunsaturated fatty acids, linoleic acid, acetoacetate and acetate	[89]
Simvastatin	Escherichia coli	*Escherichia coli* ATCC 25922 cultured on tryptic soy agar.		Cell lysate	In vitro study	GC-MS	Untargeted	Biosynthesis of amino acids, tricarboxylic acid cycle, glyoxylate shunt, glycolysis, pyruvate metabolism, purine and pyrimidine metabolisms	[94]
Statin	Human subjects who started statins and persistent nonusers during follow-up	Individuals with metabolomic profile measured at both baseline and a follow-up visit and free of statin medication at baseline.	Individuals on non-statin lipid-lowering monotherapy; Pregnant women.	Serum and plasma	Longitudinal study	NMR	Untargeted	Remnant cholesterol, omega-6 fatty acids, glycoprotein acetyl and acetate	[98]
Atorvastatin	Rats with pulmonary arterial hypertension	Sprague Dawley male rats; Weighing 200–230 g; Pulmonary arterial hypertension animal model was treated with Monocrotaline (60 mg/kg) through intraperitoneal injection.		Serum	Animal study	NMR	Untargeted	Carnitine, glucose, glycerol, acetone, leucine, isoleucine, pyruvate, acetate and choline	[99]
Pravastatin and genetic inhibition of PCSK9	Human healthy subjects	Elderly individuals at risk of cardiovascular disease with 70–82 years old; All individuals had above average plasma total cholesterol concentration (4.0 to 9.0 mmol/L) at baseline; 50% of individuals had prior vascular disease.	Pregnant women; Individuals on lipid-lowering treatment.	Serum and plasma	Randomized clinical trial (randomized placebo-controlled study vs. large population studies)	NMR	Untargeted	Lipoprotein subclasses, their lipid concentrations and composition, fatty acids, and amino acids	[108]
**PCSK9 inhibitors**									
Evolocumab	Patients with elevated Lp(a)	A selection of patients from the ANITSCHKOW trial: Male or female, ≥50 years of age at the time of informed consent; Fasting Lp(a) ≥ 125 nmol/L (50 mg/dL); Fasting LDL-C ≥ 2.6 mmol/L (100 mg/dL); For patients receiving lipid-lowering therapy, lipid-lowering therapy, including statin dose, must be unchanged for ≥8 weeks prior to screening TBRmax above 1.6 (either right carotid, left carotid or thoracic aorta) on FDG-PET/CT.	Diagnosis of diabetes mellitus or screening fasting serum glucose ≥ 7 mmol/L or glycated hemoglobin (HbA1c) ≥ 6.5%; History of homozygous familial hypercholesterolemia; Cardiovascular event within 3 months prior to randomization, or planned cardiac surgery, PCI or carotid stenting, or planned major noncardiac surgery during the course of the study period; Currently undergoing lipid apheresis; Autoimmune disease/vasculitis, active inflammatory diseases, proven or suspected bacterial infections; <1 month prior to screening or ongoing serious infection requiring intravenous antibiotic therapy; <6 weeks prior to screening or current treatment with medications that may have a significant effect on plaque inflammation; <6 weeks prior to screening or current treatment with aspirin or nonsteroidal anti-inflammatory drugs; Systemic disorders such as hepatic, renal, hematologic, and malignant diseases; History of malignancy within the last 5 years; Prior treatment with evolocumab or any other therapy to inhibit PCSK9; Pregnant or breastfeeding or planning to become pregnant or breastfeed during treatment with study drug and for an additional 15 weeks after the last dose of study drug.	Plasma	Randomized placebo-controlled clinical trial	NMR	Untargeted	VLDL, IDL and LDL particles and their lipid contents, Lp(a), fatty acids (e.g., docosahexaenoic acid)	[110]
Evolocumab	Patients with familial hypercholesterolemia	Patients with a diagnosis of familial hypercholesterolemia; Levels of LDL-C > the 95th percentile; Eligibility of patients to start treatment with PCSK-9i according to guidelines and criteria identified by Agenzia Italiana del Farmaco; All patients enrolled were on lipid-lowering therapy prior to study entry.	Age < 18 years; High level of transaminases (>3x upper normal limit); Hypertriglyceridemia (>150 mg/dL); End-stage renal failure (filtration rate <30 mL/min/m^2^); Current malignant disease or a diagnosis of malignancy in the 2 years prior to the first visit; Previous exposure to PCSK-9i; Hypercholesterolemia secondary to other causes (hypothyroidism, hormone therapies, corticosteroids, etc.).	Plasma	Interventional study	LC-MS	Untargeted	Creatine, indole, indoleacrylic acid, choline, phosphatidylcholine, and platelet-activating factor 16	[111]
Evolocumab	Patients with familial hypercholesterolemia	Patients with a diagnosis of familial hypercholesterolemia; Levels of LDL-C > the 95th percentile; Eligibility of patients to start treatment with PCSK-9i according to guidelines and criteria identified by Agenzia Italiana del Farmaco; All patients enrolled were on lipid-lowering therapy prior to study entry.	Age <18 years; High level of transaminases (>3x upper normal limit); Hypertriglyceridemia (>150 mg/dL); End-stage renal failure (filtration rate <30 mL/min/m^2^); Current malignant disease or a diagnosis of malignancy in the 2 years prior to the first visit; Previous exposure to PCSK-9i; Hypercholesterolemia secondary to other causes (hypothyroidism, hormone therapies, corticosteroids, etc.).	Serum and urine	Interventional study	LC-MS	Targeted	Small dense LDL, Lp(a), 11-dehydro-thromboxane, 8-isoprostaglandin-2alpha	[112]
**Fibrates**									
Fenofibrate	Human healthy subjects	No medication 28 days prior enrollment and during the study.		Urine (24 h)	Interventional study (fenofibrate 200 mg; 0, 7 and 14 days)	LC-MS	Untargeted	Pantothenic acid and acetylcarnitine	[114]
Fenofibrate	Mice	C57Bl/6 mice wild type and Ppara-null; Standard diet NIH31.		Urine	Animal study (0.1% fenofibrate in diet, for 7 days)	LC-MS	Targeted	Pantothenic acid and acetylcarnitine	[114]
Fenofibrate and fish oil	Mice	C57Bl/6 mice 12 weeks old.		Plasma	Animal study (0.03% fenofibrate or fish oil in diet, for 2 weeks)	LC-MS and GC-MS	Untargeted	Krebs cycle intermediates (fumaric acid, isocitric acid, malic acid, succinic acid and α-ketoglutaric acid); amino acids	[115]
Fenofibrate	Rats	Fisher 344 male rats 9 weeks old		Urine	Animal study (300 mg/kg/day fenofibrate or vehicle for 2 and 14 days)	LC-MS and GC-MS	Untargeted	Acetylcarnitine, 3-hydroxybutanoic acid, TCA cycle intermediates (i.e., malate, fumarate, alpha-ketoglutarate), glutathione metabolism (i.e., gamma glutamyltyrosine), tryptophan metabolism (kynurenine)	[113]
Fenofibrate, clofibrate, atorvastatin and pravastatin	Rats	Wistar (Crl:WI(Han)) rats in standard diet.		Plasma	Animal study: two fibrates (100 mg/kg bw/d fenofibrate, 50 mg/kg bw/d clofibrate) and two statins (70 mg/kg bw/d atorvastatin, 200 mg/kg bw/d pravastatin) in monotherapy as well as each combination of a fibrate and a statin	LC-MS and GC-MS	Untargeted	5-Oxoproline, glutamine, glycine and tryptophan	[116]
Fenofibrate and atorvastatin	Hyperlipidemic patients	Men and women with combined hyperlipidemia; >20 years; Triglyceride levels ranging from 150 to 499 mg/dL; HDL-C < 50 mg/dL; LDL-C levels requiring lipid-lowering therapy; LDL-C levels < 100 mg/dL in patients with coronary heart disease or its equivalent, including diabetes mellitus, or LDL-C levels < 130 mg/dL in all other patients, after 4 weeks of Atorvastatin 10 mg.	History of cerebrovascular or cardiovascular events in the past 3 months; Uncontrolled hypertension (systolic ≥180 mmHg or diastolic ≥110 mmHg); Uncontrolled diabetes mellitus (hemoglobin A1c levels >9%); Serum creatinine or transaminase >2× the upper limit of normal; Gall bladder disease; Thyroid dysfunction; Heavy alcohol drinking; Infection; Acute or chronic inflammatory disease; Cancer; Pregnant or breast-feeding women; History of adverse events associated with test drugs.	Serum	Randomized trial (atorvastatin escalation 20 mg vs. combined therapy, 10 mg fenofibrate and 135 mg fenofibrate, for 12 weeks)	LC-MS	Untargeted	Acylglycerols, ceramides, sphingomyelins and carnitine	[117]
Fenofibrate and simvastatin	Hyperlipidemic rats	Sprague Dawley male rats 4 months old; Normal diet and high lipid diet.		Plasma	Animal study: simvastatin (10 mg/kg daily) and fenofibrate (150 mg/kg daily) for 2 weeks	GC-MS	Untargeted	Creatinine and tyrosine	[50]
**Nutraceutical treatments**									
Annurca Apple	HuH7, hepatoma cell line			Cell lysate	In vitro study	GC-MS	Untargeted	Glutamine, acyl-carnitines, glutathione	[121]
Oat	Patients with mild cholesterol elevation	Individuals with mild hypercholesterolemia; Individuals 18 to 65 years old with BMI < 28 kg m^−2^; Total serum cholesterol values ≥ 5.18 mmol L^−1^ but ≤6.21 mmol L^−1^; TG ≤ 2.25 mmol L^−1^; No diagnoses of a serious kidney, liver, or digestive tract disease, or diabetes or other metabolic diseases; No use within the previous 3 months of relevant medicines characterized as having cholesterol-lowing effects.	Pregnancy or lactation; Daily intake of oats or other foods rich in beta-glucan for the last 6 months; History of heavy smoking or alcoholism; Current use of weight-loss diets; Poor compliance.	Serum	Randomized placebo-controlled clinical trial (40 g oats or rice twice daily (total of 80 g day−1, 3 g beta-glucan in the oats group)	LC-MS	Untargeted	Glycerophospholipid, alanine, aspartate and glutamate, sphingolipid, and retinol metabolism	[123]
*Lactobacillus plantarum* LP3	Rats	Sprague Dawley rats; Five weeks old; Weighing 120–130 g.		Cecum samples	Animal study to compare (1) normal diet (2) high-fat diet or (3) high-fat diet + L. plantarum LP3	LC-MS	Untargeted	Linoleic acid, linolenic acid and arachidonic acid	[127]
Quercetin and resveratrol	Mice treated with high-fat diet	C57/6J mice 7 weeks old.		Liver tissue	Animal study to compare normal diet (Normal) group fed with normal diet; high-fat diet, for 26 weeks (HFD) group; quercetin (Quercetin) group fed with HFD and supplemented with 0.4% quercetin (4 g/kg diet); resveratrol(Resveratrol) group fed with HFD and supplemented with0.4% resveratrol (4 g/kg diet); combined quercetin and resveratrol (Combined) group fed with HFD and supplemented with 0.2% quercetin and 0.2% resveratrol (2 g quercetin + 2 g resveratrol per kg diet)	GC-MS	Untargeted	4-aminobutiric acid, ornithine and histidine	[129]

BMI, body mass index; CAD, coronary artery disease; CE-MS, capillary electrophoresis-mass spectrometry; FDG, fluorodeoxyglucose; FH, familial hypercholesterolemia; GC-MS, gas chromatography-mass spectrometry; HDL, high-density lipoprotein; IDL, intermediate-density lipoprotein; LC-MS, liquid chromatography-mass spectrometry; LDL-C, low-density lipoprotein cholesterol; Lp(a), lipoprotein(a); NMR, nuclear magnetic resonance; NSTEMI, non ST-segment elevation myocardial infarction; PCSK9, proprotein convertase subtilisin/kexin type 9; PET/CT, positron emission tomography/computed tomography; STEMI, ST elevation myocardial infarction; TG, triglycerides; VLDL, very-low-density lipoprotein.

## Abbreviations

AAE	Annurca apple polyphenolic extract
ACS	acute coronary syndrome
ASCVD	atherosclerotic cardiovascular disease
ATP	adenosine triphosphate
CAP	Cholesterol and Pharmacogenetic
CE	capillary electrophoresis
CPT-1	carnitine palmitoyltransferase I
CVD	cardiovascular disease
FH	familial hypercholesterolemia
FT-ICR	Fourier transform-ion cyclotron resonance
GC	gas chromatography
GlycA	glycoprotein acetylation
GSK-3β	glycogen synthase kinase-3β
HDL	high-density lipoprotein
HK-2	hexokinase 2
HMG-CoA	3-hydroxy-3-methyl-glutaryl-coenzyme A
IDL	intermediate-density lipoprotein
LC	liquid chromatography
LDL	low-density lipoprotein
LDL-C	low-density lipoprotein cholesterol
Lp(a)	lipoprotein(a)
MRM	multiple reaction monitoring
MS	mass spectrometry
NIST	National Institute of Standards and Technology
NMR	nuclear magnetic resonance
NO	nitric oxide
NOS	nitric oxide synthase
PAH	pulmonary arterial hypertension
PCSK9	proprotein convertase subtilisin/kexin type 9
PPARα	peroxisome proliferator-activated receptor alpha
PUFAs	polyunsaturated fatty acids
Q-TOF	quadrupole-time of flight
ROS	reactive oxygen species
SNP	single nucleotide polymorphism
SREBP-1c	sterol regulatory element-binding protein 1c
TC	total cholesterol
TGs	triglycerides
TOF	time of flight
UHPLC	ultra-high-performance liquid chromatography
UPLC	ultra-performance liquid chromatography
VLDL	very-low-density lipoprotein
WHHL	Watanabe heritable hyperlipidemic
